# Assessment of Volume Status and Fluid Responsiveness in Small Animals

**DOI:** 10.3389/fvets.2021.630643

**Published:** 2021-05-28

**Authors:** Søren R. Boysen, Kris Gommeren

**Affiliations:** ^1^Department of Veterinary Clinical and Diagnostic Sciences, Faculty of Veterinary Medicine, University of Calgary, Calgary, AB, Canada; ^2^Department of Companion Animals, Faculty of Veterinary Medicine, University of Liège, Liège, Belgium

**Keywords:** dogs, cats, fluid responsiveness, volume status, dynamic, static, POCUS, wet lung

## Abstract

Intravenous fluids are an essential component of shock management in human and veterinary emergency and critical care to increase cardiac output and improve tissue perfusion. Unfortunately, there are very few evidence-based guidelines to help direct fluid therapy in the clinical setting. Giving insufficient fluids and/or administering fluids too slowly to hypotensive patients with hypovolemia can contribute to continued hypoperfusion and increased morbidity and mortality. Similarly, giving excessive fluids to a volume unresponsive patient can contribute to volume overload and can equally increase morbidity and mortality. Therefore, assessing a patient's volume status and fluid responsiveness, and monitoring patient's response to fluid administration is critical in maintaining the balance between meeting a patient's fluid needs vs. contributing to complications of volume overload. This article will focus on the physiology behind fluid responsiveness and the methodologies used to estimate volume status and fluid responsiveness in the clinical setting.

## Introduction

Although the route of administration is not always specified within this review, it should be presumed all references to fluids refer to intravenous (IV) administration unless otherwise stated. In hemodynamically unstable patients, IV fluids are administered to increase cardiac output (CO) and improve tissue perfusion (1). However, in humans, cats, and dogs both insufficient and excessive IV fluid administration are associated with increased morbidity and mortality (2–8). In human medicine, recommendations have historically focused on the rapid and efficient administration of fluids (referred to as “fluid loading”), however, over the past few decades mounting evidence has demonstrated the negative effects of overzealous fluid therapy (“fluid overload”) (5, 6, 9–12). As a result, a clear distinction between two important aspects of fluid resuscitation has recently emerged: (1) assessment of volume status and (2) assessment of fluid responsiveness. Volume status attempts to determine if a patient's circulatory volume is decreased, normal, or increased at a static point in time. Volume responsiveness on the other hand considers a dynamic question; will additional fluid administration lead to an increase in stroke volume (SV) and hence CO. This review offers an overview of the pathophysiology and monitoring of fluid administration, with an emphasis on fluid responsiveness.

## Fluid Responsiveness

**Figure 1:**
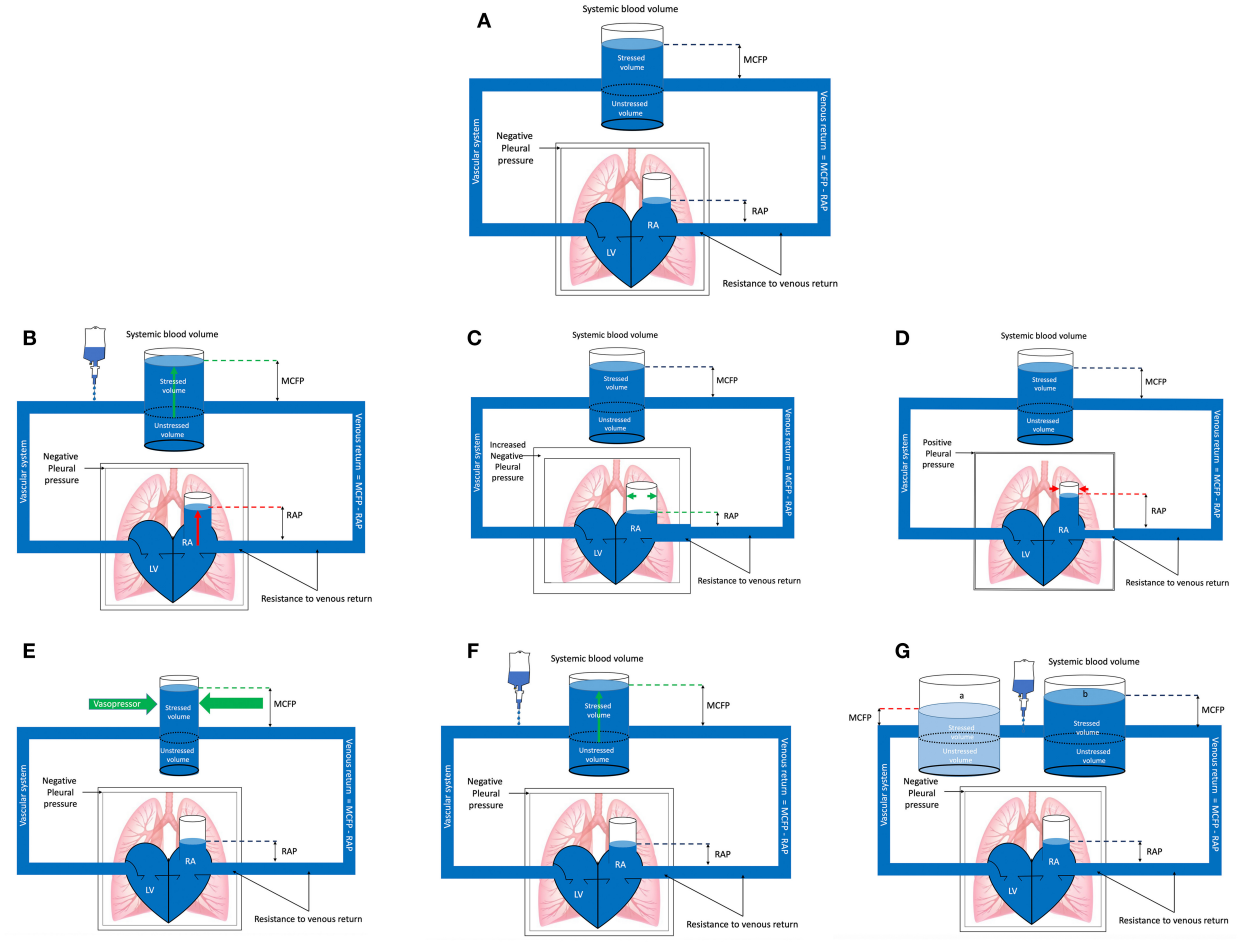
**(A)** Baseline. The pressure gradient between the right atrium (RA) and mean circulatory filling pressure (MCFP) determines venous return (VR) or preload. The veins are capacitance vessels. The blood volume contained within the venous system that does not contribute to pressure or stress being applied to the vessel wall is referred to as the unstressed volume. This unstressed blood volume depends on vessel size (and thus venoconstriction or -dilation). Any additional blood volume added to the venous system beyond the unstressed volume will exercise a force on the wall of the vein, distending it, thus generating a transmural pressure above zero. This additional blood volume is referred to as the stressed volume and is the main contributor to MCFP. Also depicted is the negative pleural pressure which changes during the respiratory cycle and influences the heart and VR, referred to as heart-lung interactions. RAP, right atrial pressure; LV, left ventricle. **(B)** Spontaneous inspiration. In the spontaneously breathing patient negative pleural pressure increases during inspiration, decreasing the resistance to venous return (VR) and the right atrial pressure (RAP). This results in a greater difference between the mean circulatory filling pressure (MCFP) and RAP, subsequently increasing VR or preload. The opposite effect occurs during expiration. All other factors held constant, the magnitude of the effect and impact on VR will vary depending on the volume status of the patient: The more hypovolemic the patient is, the greater the result of heart-lung interactions on VR. RA, right atrium; LV, left ventricle. **(C)** Mechanical inspiration. During the inspiratory phase of positive pressure ventilation the pleural pressure rises (becomes less negative) which increases the resistance to venous return and directly compresses the right atrium, increasing the right atrial pressure (RAP). This results in a decrease in the difference between the mean circulatory filling pressure (MCFP) and RAP, subsequently decreasing venous return (VR) or preload. The opposite effect occurs during expiration. All other factors held constant, the magnitude of the effect and impact on VR will vary depending on the volume status of the patient: The more hypovolemic the patient is the greater the result of heart-lung interactions on VR. RA, right atrium; LV, left ventricle. **(D)** Vasopressor administration. Administration of a vasopressor causes vasoconstriction which, all other factors held constant, may result in an increase in the stressed blood volume relative to the unstressed blood volume, depending on the type of vasopressor administered (arterial, venous or mixed). The increase in stressed volume will increase the mean circulatory filling pressure (MCFP), increasing venous return (VR). This partly explains the improvement in cardiac output (CO) sometimes seen with vasopressor administration. However, because vasopressors will also increase resistance to venous flow (increase not illustrated in this diagram), vasopressors may only cause a minimal increase in VR. RAP, right atrial pressure; RA, right atrium; LV, left ventricle. **(E)** Fluid loading, fluid responsive. For fluid loading to be effective it must increase the stressed volume more than it increases right atrial pressure (RAP). In this illustration fluid loading has caused a larger increase in the stressed volume with minimal increase in RAP. Therefore, the mean circulatory filling pressure (MCFP) increases while the RAP is minimally increased, and the result is an increase in venous return (VR). Understanding the Frank Starling curve helps explain if the stressed volume increases more than the unstressed volume or RAP. RAP, right atrial pressure; RA, right atrium; LV, left ventricle. **(F)** Fluid loading, fluid unresponsive. In this example a fluid bolus is administered which increases the stressed blood volume and mean circulatory filling pressure (MCFP), however, at the same time there is a parallel increase in right atrial pressure (RAP). The net results is a failure of venous return (VR) and subsequently cardiac output (CO) to increase (fluid unresponsive). Given organ blood flow is driven by the difference between MAP and central venous pressure (CVP), and that CVP becomes the major factor determining organ and microcirculatory flow when MAP is within an organ autoregulatory range, an increase in CVP may contribute to organ injury. **(G)** Fluid loading, fluid unresponsive: In this example, all other factors held constant, vasodilation causes an increase of the unstressed blood volume relative to the stressed volume [**(a)**, light blue], which decreased the mean circulatory filling pressure (MCFP) (red dotted line). All other factors held constant, fluid loading **(b)** increases the stressed volume and returns the MCFP to baseline (blue dotted line) but has failed to increase cardiac output (CO) because insufficient fluids have been administered to increase MCFP above baseline. With vasodilation the proportion of unstressed volume increases relative to the stressed volume, resulting in a greater volume of fluid needing to be administered before a significant increase in the stressed volume is noted. The right atrial pressure (RAP) will also change with alterations in the stressed blood volume and vasodilation, which has not been illustrated here for simplicity. Changes in vascular tone because of fluid loading are also not shown for simplicity. Understanding the Frank Starling curve helps explain if the stressed volume increases more than the unstressed volume or RAP. RA, right atrium; LV, left ventricle.

A consensus on the exact definition of fluid responsiveness is lacking. In general, it can be considered an attempt to identify patients who will have a positive physiologic response to fluid administration. More precisely it can be defined as “*the positive reaction of a physiologic parameter of a certain size to a standardized volume of a certain type of fluid (or other type of induced preload change) within a certain amount of time and measured within a certain interval”* (13). Unfortunately, defining fluid responsiveness is complicated by a lack of consensus regarding the ideal physiologic parameter(s) to measure, the degree of change in the measured physiologic variable that defines a positive response, what defines a preload challenge, and if an IV fluid bolus is used as the preload challenge, the amount of fluid that defines a standardized volume. In general, the physiologic parameter measured can be classified as either static or dynamic depending on how it is measured, which is explained in detail in section Defining Static vs. Dynamic Markers below. In both the human and veterinary literature, the magnitude of change (increase or decrease) that constitutes a positive response varies from 6 to 36% depending on the physiologic parameter measured and conditions under which the preload challenge is induced (14–27). In general, there is a trend to define a positive fluid responder as any patient that has an increase in a measured dynamic marker, such as CO, by ≥10–15% following a preload challenge (14, 16, 25, 28–30). There are generally two ways to induce a preload challenge; (1) by directly increasing the vascular volume through a traditional or a mini IV fluid bolus, or (2) shifting fluids within the vascular system without actually changing the patients total vascular volume. The latter is accomplished through maneuvers such as passive leg raising (PLR), or by assessing the effect of inspiratory and expiratory pressures on venous return (VR). PLR maneuvers, which are often used in human ICU patients, shift blood from the periphery to the central circulation increasing VR without actually administering fluids to the patient (26, 29). Although PLR maneuvers have been investigated in anesthetized swine, research on its application in cats and dogs is lacking (31). Manipulating respiratory pressures in patients receiving positive pressure ventilation influences heart-lung interactions (see Figures 1A–C), which can impact VR without changing the total circulating blood volume (26, 29). These concepts are discussed in greater detail in section Fluid Bolus, Mini Bolus, Heart-Lung Interactions and Passive Leg Raising. In the veterinary clinical setting, a preload challenge is most often elicited through a traditional IV fluid bolus, typically described as isotonic fluids at a dose of 10–20 mL/kg administered over a period of 10–15 min in both cats and dogs (14, 32–34). More recently, and in many veterinary research settings, a mini fluid bolus which is often defined as 3–5 ml/kg IV isotonic fluid administered over 1 min (15, 16, 20, 23, 35) has been used to induce a preload challenge. However, in clinical practice there will be considerable variation in the volume of fluid administered because of clinician preference, patient characteristics such as species and age, the type of fluid used, and the underlying disease process. Regardless of what techniques are used the goal of fluid responsiveness is to determine if a patient will benefit from additional IV fluid administration while minimizing the risk of fluid overload. Therefore, fluid responsiveness can be summarized as the presence or absence of a positive measurable reaction following the administration of a precise preload challenge.

To understand fluid responsiveness, it is important to differentiate the concepts of fluid loading and a preload challenge. Fluid loading is the rapid administration of IV fluids for suspected hypovolemia, often in the absence of monitoring a real time response to fluid administration (13, 36, 37). It is most often applied in the emergency and critical care (ECC) setting when confronted with severe life-threatening hypotension and hypoperfusion secondary to overt hypovolemia. In contrast, a preload challenge is a test of the cardio-circulatory system designed to assess if a patient has preload reserve that will increase CO with additional IV fluid administration (fluid responsiveness) (36). It allows more individualized patient fluid therapy, which may be preferred over protocolized therapy in some settings (36).

Although volume status and fluid responsiveness are often considered simultaneously, they should be evaluated independently in individual patients. Many hypovolemic patients require fluid bolus resuscitation to improve tissue perfusion and reverse the potentially fatal consequences of shock. In the human intensive care unit (ICU) setting, 20% of patients receive resuscitation fluids, with this number increasing to >30% on the first day of ICU admission (1). Results are likely similar or even higher in veterinary ICU patients. Common reasons for administration of bolus fluid therapy include reversal of severe hypovolemia, sepsis, large perioperative volume losses, hemodynamic derangements and oliguria that is volume responsive (32, 38, 39). Although the number of ICU patients receiving bolus fluid resuscitation has remained fairly constant over the past decade, the rationale for initiation of IV fluid bolus therapy has shifted in human ICUs; more patients are receiving therapy in response to signs of impaired tissue perfusion or a measured decrease in CO, with fewer patients receiving therapy in response to abnormal vital signs in the absence of signs of abnormal perfusion (1). This emphasizes a paradigm shift away from “a one size fits all” and the practice of “volume loading,” to establishing “euvolemia” and an “optimal fluid balance,” particularly in patients with systemic inflammation or sepsis.

Merely assessing volume status does not predict a patient's response to fluid loading. A patient may be hypovolemic and non-fluid responsive due to the complex interaction of the glycocalyx, vascular permeability, capillary leak, vascular tone, and cardiac function. At the same time, the fact that a patient is fluid responsive does not always imply hypovolemia is present. For example, healthy euvolemic patients given a fluid bolus may meet the definition of being fluid responsive. Finally, a patient that is hypovolemic and fluid responsive may still develop complications of volume overload, particularly if increased vascular permeability is present (40, 41). For example, due to pre-existing or fluid induced endothelial dysfunction, it is reported that within an hour <5% of a fluid bolus remains within the IV space of some human patients with sepsis (41). In patients that are predisposed to volume overload the prediction of fluid responsiveness is vital in trying to determine if inotropic and/or vasopressor support is preferred over fluid replacement.

Given there is a lack of data regarding evidence-based fluid management in hypotensive human and veterinary ECC patients it is not surprising there is considerable heterogeneity in the way IV fluids are administered in shock states, including the criteria used as triggers, targets, volume, and safety limits for fluid input (31, 36, 39, 42). Much of the heterogeneity likely stems from diverse backgrounds of professionals involved in initial resuscitation, a clear lack of evidence-based guidelines, the different pathophysiological processes causing hypotension, and the reliance on simple clinical physiological variables to estimate fluid responsiveness. The remainder of this article will explain the pathophysiological processes of fluid responsiveness, after which monitoring tools for volume status and fluid responsiveness will be discussed, and the heterogeneity in how fluid responsiveness is assessed in veterinary medicine will be highlighted.

## The (Patho)Physiology of Fluid Responsiveness

Understanding circulatory physiology and pathophysiology helps understand how different scenarios mandate different therapeutic and fluid management strategies. Two general assumptions are expected in response to a preload challenge; (1) increased venous volume leads to increased cardiac preload (venous loading = cardiac loading), (2) increased preload leads to increased stroke volume/cardiac output (VR = CO).

### Mean Circulatory Filling Pressure: Increased Venous Volume Leads to Increased Cardiac Preload (Venous Loading = Cardiac Loading)

It may seem intuitive that an IV fluid bolus will lead to increased VR. However, a clear understanding of circulatory physiology may explain why increased VR is not always observed.

Preload, or VR, is determined by the pressure gradient between capacitance veins and the right atrium (RA). The pressure within the capacitance veins is termed mean circulatory filling pressure (MCFP) (13, 29, 30). The venous system is composed of highly distensible capacitance veins, which contain the majority of blood volume (13, 29). These capacitance veins require a minimal volume of blood to prevent them from collapsing, which occurs if transmural pressure becomes negative. This “minimal” blood volume to prevent collapse depends on vessel size (and thus venoconstriction or -dilation) and is referred to as the unstressed volume; it is any blood volume that can be added to the venous system that does not contribute to pressure or stress being applied to the vessel wall above a transmural pressure of zero (13, 29, 30). Note that venous pressure is the pressure created by venous blood against the wall of the vein (intraluminal) regardless of the pressure surrounding the vessel, while transmural pressure is the difference in pressure across the wall of a vessel (within the vessel and outside the vessel). Any additional blood volume added to the venous system beyond the unstressed volume will exercise a force on the wall of the vein, distending it, thus generating a transmural pressure above zero (13, 29, 30). This additional blood volume is referred to as the stressed volume and is the main contributor to MCFP (Figure 1A) (29). Think of a horizontally placed water-filled balloon, in which you create a tiny hole in one end. The water will leak out of the balloon until the transmural pressure becomes zero, however, there will still be a residual amount of water present inside the balloon. This amount of fluid, which does not stretch the wall of the balloon above a transmural pressure of zero, would be considered unstressed volume and does not contribute to fluid movement out of the balloon. The volume of water that poured out of the balloon was the stressed volume, as it stretched the wall of the balloon creating a force (transmural pressure above zero) that acted to move fluid out of the balloon.

MCFP [or mean systemic pressure (MSP)] varies with changes in venous blood volume or vessel tone, which determines the balance of venous blood contained within the unstressed and stressed blood volume. As a consequence, MCFP may only be increased by changes in blood volume, or by changes in vessel tone (Figures 1D–G) (29). This partly explains the improvement in CO sometimes seen with vasopressor administration (29).

MCFP is predominantly regulated by the effects of the sympathetic nervous system on the splanchnic venous system (43). Depending on the species, the splanchnic venous system contains 20–30% of the total blood volume, is 30 times more compliant than the arterial circulation, and is heavily innervated with α-adrenoceptors (43). It serves as a reservoir of blood contained within capacitance vessels which can easily change in volume to maintain VR to the heart (13).

MCFP, right atrial pressure (RAP) and the resistance to venous flow (Rv) are the main driving forces of VR as expressed through the following formula: VR = [(MCFP – RAP)/Rv], (Figure 1A) (13). This formula implies VR can be increased by one of 3 mechanisms; (1) lowering RAP, (2) decreasing Rv, or (3) increasing MCFP (Figures 1B–G).

From a physiologic standpoint, given VR is determined by the pressure gradient between capacitance veins and the right atrium, decreasing the right atrial pressure should increase VR, provided all other variables are held constant. However, techniques to decrease RAP are limited, and if RAP falls too low, venous collapse at the thoracic inlet may occur (13). Venous collapse causes an increase in Rv and subsequently a decrease in VR. Therefore, in the clinical setting it is generally not practical to decreases RAP.

Resistance to venous flow may be decreased by increasing venous compliance through venodilation (35). However, venodilation results in an increase in the ratio of the unstressed to stressed blood volume and a subsequent decrease in MCFP. Therefore, depending on the relative changes in the Rv and MCFP, the net effect on VR is uncertain.

**Figure 2:**
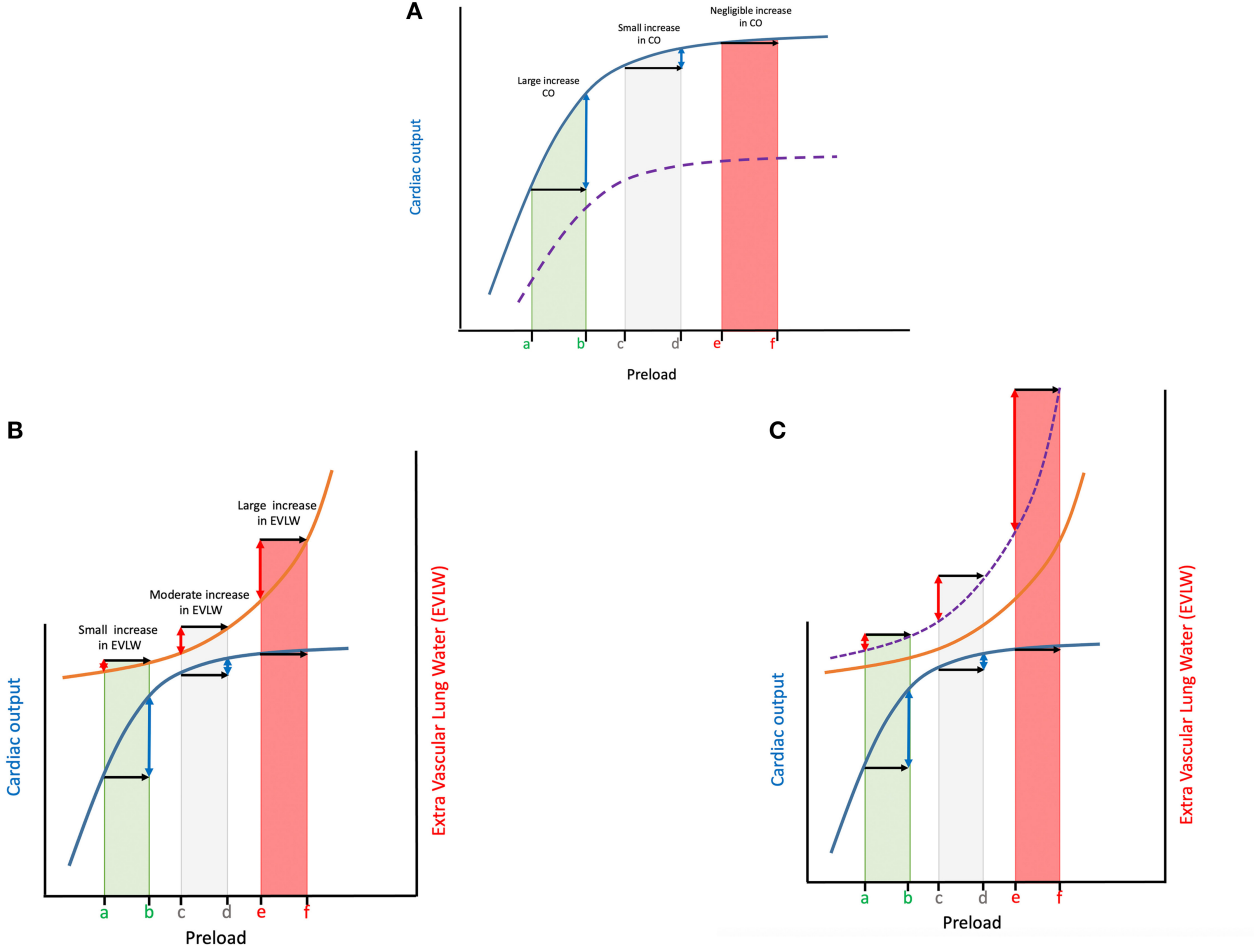
**(A)** The Frank Starling curve is shown in blue for 3 different patients with a similar Frank-Starling curve (blue line), yet located at different points along that curve; The x axis shows an induced increase in preload (e.g., fluid bolus) of the same magnitude for all 3 patients (black arrows). As a result of the shape of the curve the change in cardiac output (CO) depicted on the y-axis (blue double-headed arrows) is very different depending on the point along the curve the patient is located. Patient a-b has a very large increase in CO relative to patient c-d (minimal increase in CO) or patient e-f (negligible change in CO). In patient a-b the increased preload leads to stretching of the myocytes, which subsequently contract more forcefully, leading to an increase in CO. For a patient to be fluid responsive both the right and left ventricles must be functioning on the steep part of the Starling curve. Many critically ill human patients, and most likely a proportion of our companion animals have significant changes in cardiac contractility (e.g., due to acidosis, sepsis, etc.), flattening the curve and shifting it to the right (purple dotted line), placing them on the flat portion of the curve and decreasing the rise in CO for a similar rise in preload. **(B)** The Marik-Philips curve (orange line) is superimposed on the Frank-Starling curve (blue line) to show the relationship between preload changes, cardiac output (CO) and extravascular lung water (EVLW). A patient located at the left of the curve (patient a-b), is on the steep portion of the Frank-Starling curve, has low preload prior to fluid therapy, and is very likely to be fluid responsive. This implies a fluid bolus would increase preload (black arrow), leading to an increase in CO (blue double-headed arrow). At the same time, according to the Marik-Philips curve, this increase in preload would not be associated with a significant increase in extravascular lung water (EVLW; red double-headed arrow). A patient located on the right side of the curve (patient e-f), has a relatively high preload prior to fluid therapy. Providing an additional fluid bolus to increase preload the same amount as patient a-b will have little to no effect, with no improvement in CO, but a very significant increase in EVLW. Between patient a-b and e-f is the “gray zone” where it is far more difficult to predict the impact of fluid boluses on preload, CO and EVLW (patient c-d). See text regarding gray zone effect. **(C)** The Marik-Philips and Frank-Starling curve for patients in septic shock where the Frank-Starling curve is flattened and reaches a plateau more quickly (not shown) while the Marik-Phillips curve is shifted to the left (purple dashed line) due to changes in vascular permeability, lung compliance, glycocalyx derangements, etc. Septic shock patients are more often located on the flat portion of the Frank-Starling curve and the steeper portion of the Marik-Phillips curve (patient e-f), particularly following initial fluid resuscitation. These patients are more likely to benefit from vasopressors and positive inotropes as additional fluid boluses to increase preload (black arrows) will not increase cardiac output (CO) (blue double-headed arrows) and will likely lead to volume overload and increased extravascular lung water (EVLW) (double-headed red arrows). This is because of the curvilinear shape of the left ventricular pressure-volume curve and the result of altered diastolic compliance at higher filling pressures. As atrial pressure increases, venous and pulmonary hydrostatic pressures increase, resulting in the release of natriuretic peptides. These natriuretic peptides will cause fluids to shift from the vascular space into the interstitial space. The shift of fluids to the interstitial space results in pulmonary and peripheral tissue edema. Septic patients have a higher tendency to accumulate EVLW (shift of Marik-Phillips curve to the left), and thus fluid administration should be titrated more carefully in such patients.

**Figure 3:**
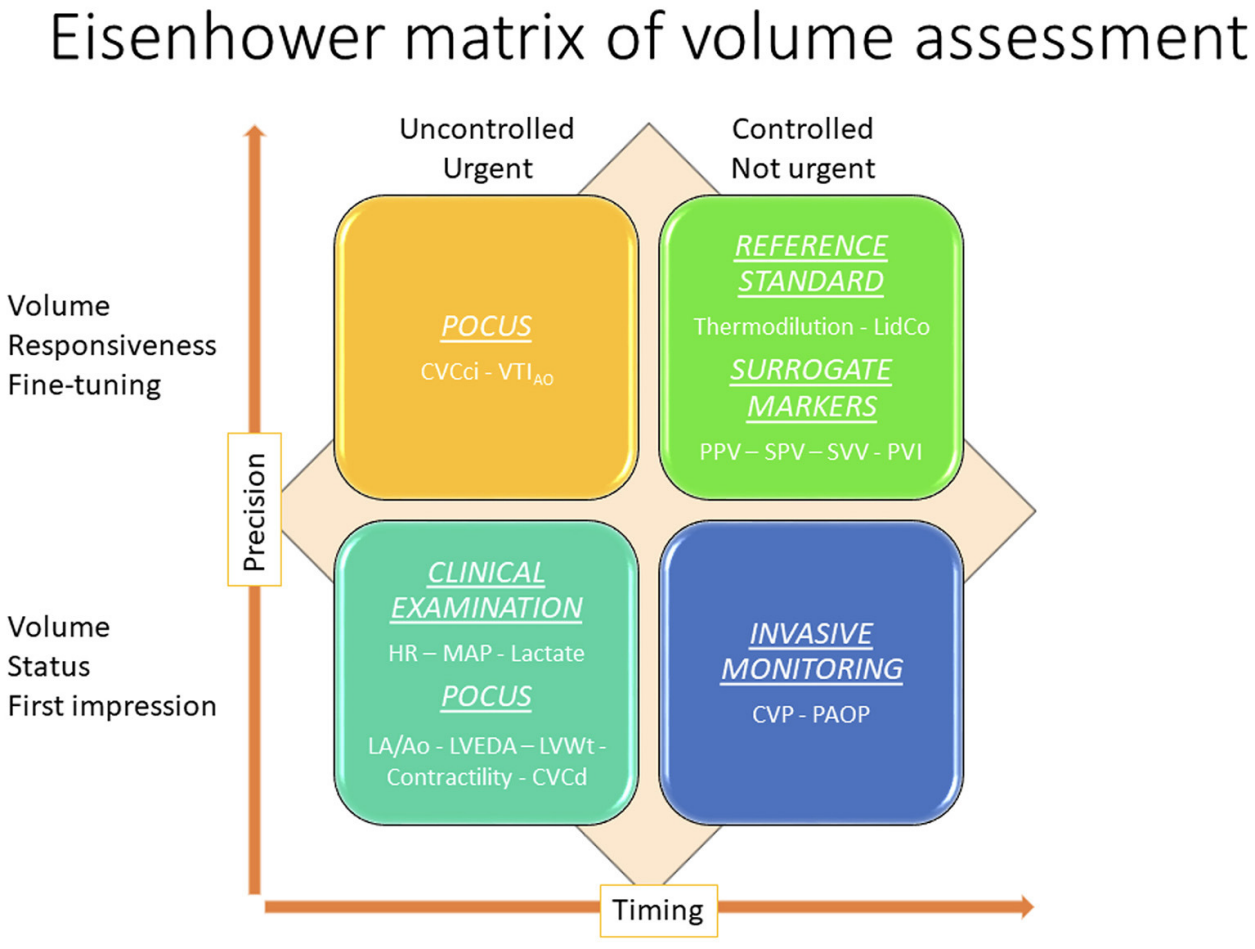
Eisenhower matrix of volume assessment demonstrating how the application of different volume assessment techniques are used to assess volume status and fluid responsiveness, the relationship between the techniques, and the relative degree of environmental control, degree of precision, and timing for each. CVC, caudal vena cava; PPV, pulse pressure variation; SPV, systolic pressure variation; SVV, stroke volume variation.

MCFP can be increased through venoconstriction or fluid loading (Figures 1D,E) (13, 29, 44, 45). Venoconstriction, because it will increase Rv, may only cause a minimal increase in VR. Fluid loading is therefore often used to try and increase VR, however, for fluid loading to be effective it must increase the stressed volume to a greater degree than it increases the RAP (Figure 1E) (45). Fluid loading may fail to increase CO when: (1) patients' have a parallel increase in RAP and MCFP (Figure 1F), (2) MCFP fails to increase due to inadequate volume being administered to vasodilated patients (Figure 1G), and (3) patients are preload unresponsive (on the flat portion of Frank-Starling curve (Figure 2A) (36, 45). Organ blood flow is driven by the difference between mean arterial pressure (MAP) and RAP in contrast to VR which is driven by the difference between MCFP and RAP. Therefore, any increase in RAP can contribute to decreased organ blood flow if MAP remains constant. Given RAP is the major factor determining organ and microcirculatory flow when MAP is within an organ's autoregulatory range, an increase in RAP may contribute to organ injury (45). Understanding the Frank-Starling curve helps explain what might occur if the stressed volume increases more than RAP.

### Frank-Starling and Marik-Philips Curve: Increased Preload Leads to Increased Stroke Volume/Cardiac Output (Venous Return = Cardiac Output)

The Frank-Starling curve (Figure 2A) describes the relationship of preload and SV, which subsequently determines CO and oxygen delivery (DO_2_) to the tissues. The relationship between these parameters can be expressed through the following 2 formulas: (1) DO_2_ = CaO_2_ × CO, and (2) CO = HR × SV. Moreover, MAP = CO × SVR.

Where DO_2_ is oxygen delivery (mL/min), CaO_2_ is the arterial oxygen content (mL/dL), CO is cardiac output (mL/min), HR is heart rate (beats/min), SV is stroke volume (mL), MAP is mean arterial pressure (mmHg), and SVR is systemic vascular resistance (dynes/seconds/cm^−5^).

Stroke volume is the amount of blood ejected from the heart with each beat and is dependent on preload (end-diastolic wall tension), contractility and afterload (end-systolic wall tension). According to the Frank-Starkling curve, within certain limits an increase in VR will lead to an increase in preload (29). This increased preload leads to stretching of the myocytes, which subsequently contract more forcefully, leading to an increase in SV (Frank-Starling law). *This is the foundation for the concept of fluid responsiveness*.

For a patient to be fluid responsive both the right and left ventricles must be functioning on the steep part of the Frank-Starling curve (wherein increased filling improves CO, without any major changes in other determinants of CO, such as contractility, afterload and diastolic dysfunction) (29).

Cardiac contractility can be affected by a variety of processes, fluid overload and myocardial hibernation to name a few. During fluid overload, myocytes get stretched beyond a certain level, rendering them unable to contract more forcefully, with SV subsequently failing to increase any further (fluid “unresponsiveness”). At this point fluid administration is likely to increase the stressed volume and thus MCFP, but to a lesser extent than it increases RAP, leading to volume overload (Figures 2A–C) (44). In addition, many critically ill human patients, and most likely a proportion of companion animals, have significant changes in cardiac contractility (e.g., due to acidosis, sepsis, etc.) placing them on the flat portion of the curve. Finally, an increase in afterload (e.g., due to vasoactive agents) can also decrease CO (44).

The Marik-Philips curve describes the relationship between increasing preload and extravascular lung water (EVLW). When the Marik-Philips curve is superimposed over the Frank-Starling curve the risk of a patient developing edema during fluid loading is nicely illustrated (Figure 2B) (45). A patient located at the left of the curve (the steep portion of the Frank Starling curve), has low preload prior to fluid therapy, and is very likely to be fluid responsive. This implies a fluid bolus would increase preload, leading to an increase in CO. At the same time, according to the Marik-Philips curve, this increase in preload would not be associated with a significant increase in EVLW (45). A patient located on the right side of the curve (the flat portion of the Frank-Starling curve), has a relatively high preload prior to fluid therapy. Providing an additional fluid bolus will have little to no effect on preload, and thus no improvement in SV, but is likely to have a significant increase in EVLW.

It should be remembered that every patient has its own unique set of individual Frank-Starling and Marik-Philips curves, and that these curves are influenced by factors associated with specific disease states. Knowing the factors that govern these equations will allow the clinician to estimate the effect of fluid administration, but should never be considered an absolute certainty.

The underlying cause of shock will influence an individual patient's Frank-Starling and Marik-Philips curves, and subsequently fluid responsiveness. The incidence of hypotension in hemodynamically unstable human ICU patients is as high as 33%. Considering Weil's classification of shock, it is reported in humans that 62–71% of cases are due to septic shock, 16% cardiogenic, 16% hypovolemic, 4% other distributive shock, and 2% obstructive shock (46). In contrast, the incidence of hypotension due to sepsis in small animal ICU patients, although not well-documented, appears lower. A canine study of 35 dogs listed causes of hypovolemia (46%) more commonly than causes of sepsis (29%) in dogs with confirmed hypotension (32). Similarly, a feline study of 39 cats with hypotension found cases with suspected hypovolemia (renal or urinary disease 21%, gastrointestinal disease 13%) to be more frequent than cases with suspected sepsis, which was only documented in a small percentage of cases (39). As the Frank-Starling and Marik-Philips curves are very different between hypovolemic and septic patients, the clinical question asked (volume status or fluid responsiveness) will be impacted by the type and severity of shock.

Hypovolemic shock patients are usually located on the ascending portion of the Frank Startling curve (fluid responders). These patients are likely to benefit from fluid administration. Administration of fluid boluses will increase SV, which typically results in improved clinical cardiovascular parameters such as HR and MAP. At the same time, their Marik-Philips curve will not be as steep, and the administered fluid boluses are unlikely to cause a dramatic increase in EVLW (Figures 2B,C). This combination results in a clinical scenario where the safety margins are relatively large (45). However, monitoring volume status is still recommended in patients presenting with hypovolemic shock that initially respond to fluid resuscitation, as research in humans suggests patients with persistent parameters indicative of hypovolemia, despite normalization of blood pressure and vital signs, may be at increased risk of relapsing into a state of shock (47).

Septic shock patients are more often located on the flat portion of the Frank-Starling curve (non-fluid responders), particularly following initial fluid resuscitation. These patients are more likely to benefit from vasopressors and positive inotropes as additional fluid boluses will not increase SV and will likely lead to volume overload and increased EVLW (45). This is because of the curvilinear shape of the left ventricular pressure-volume curve and the result of altered diastolic compliance at higher filling pressures. As atrial pressure increases, venous and pulmonary hydrostatic pressures increase, resulting in the release of natriuretic peptides. These natriuretic peptides, along with the increased hydrostatic pressure, will cause fluids to shift from the vascular space into the interstitial space. The shift of fluids to the interstitial space results in pulmonary and peripheral tissue edema. Tissue edema contributes to decreased oxygen and metabolite diffusion, distorts tissue architecture, impedes capillary blood flow and lymphatic drainage and disturbs cell-cell interactions. Septic patients have a higher tendency to accumulate EVLW, and thus fluid administration should be titrated more carefully in such patients. With sepsis, not only are patients less likely to be fluid responsive, but the Marik-Philips curve is also shifted to the left, further increasing the risk of volume overload and increased EVLW (Figure 2C) (45).

As the majority of ECC veterinary patients have non-distributive hypovolemic shock, they are more likely to respond to volume resuscitation (32, 39) and less likely to experience volume overload than human ICU patients in shock (46). In contrast, the majority of hypotensive human ICU patients have distributive septic shock with complex hemodynamic derangements that can significantly affect homeostasis of the cardiovascular system (e.g., low oncotic pressure, decreased glycocalyx integrity, increased permeability, vasodilation, etc.) (46). This may partially explain why hypotensive dogs are more likely to respond to fluid bolus therapy than human ICU patients.

Roughly 50% of preload challenges performed in critically ill human patients do not result in an increase in CO or SV, exposing these patients to the potential harm of fluid overload (48). Research regarding fluid responsiveness in small animals is sparse. A single center retrospective study consisting of 35 dogs demonstrated 60–65% of dogs that present to the ECC service with hypotension (Doppler blood pressure <90 mmHg) will respond to fluid bolus therapy, defined as normalization of blood pressure (32). Although response in hypotensive dogs did not vary with the underlying cause, sample size was small and the sensitivity of using normalization of blood pressure (a marker of volume status rather than of fluid responsiveness), may have failed to detect some fluid responsive dogs. The actual value of hypotensive dogs responding to fluids may be higher or lower than 65% depending on what physiologic parameters are measured and how a response to fluid therapy is defined. Larger prospective canine and feline studies are required to further explore these findings.

A major clinical challenge that all veterinarians must address when confronted with a hemodynamically unstable patient is to successfully identify patients that will increase CO in response to fluid therapy (fluid responsive) vs. those who will develop adverse events from unnecessary administration of IV fluids (non-fluid responsive). The previous paragraph indicates not every patient, human or veterinary, will be a fluid responder. In severely hypovolemic patients additional fluid boluses have a high benefit to risk ratio (as stated above, volume status is the focus over fluid responsiveness in the resuscitation phase of fluid therapy). Conversely, additional fluid boluses will be detrimental in markedly hypervolemic patients. Between the extremes of severe hypovolemia and marked hypervolemia, predicting if the patient will benefit from fluid therapy without developing complications of fluid overload (e.g., the patient is fluid responsive and fluid tolerant) is more challenging. Answering whether an additional fluid bolus will be beneficial (improve CO) or not (cause volume overload) with 100% accuracy is not always possible. The gray-zone approach, which is a form of statistical analysis, suggests a “three level decision tree” when assessing if a patient might be fluid responsive or not: “Yes—Maybe—No.” The gray-zone approach can be calculated for any physiologic parameter used to assess a patient's response to a preload challenge (e.g., heart rate, CO, SV, etc.). More specifically, it is defined as the interval of values (for the physiologic parameter assessed) between a sensitivity or specificity lower than 90% to classify patients as fluid responders or non-responders (16, 17, 19). The further from the gray zone a measurement lies, the greater the confidence of accurately classifying the patient as a fluid responder or not. Values within the gray zone do not allow the patient's response to a preload challenge to be determined with a high degree of confidence. When a patient lies within the gray-zone, meaning it is not easy to determine if the parameter assessed will predict fluid responsiveness, other available clinical parameters should be assessed to guide fluid strategies, ideally performed serially to track changes over time.

## Assessing Fluid Responsiveness

### Defining Static vs. Dynamic Markers

Static markers, such as central venous pressure (CVP), MAP, inferior vena cava (IVC) diameter, and end-diastolic ventricular area (LVEDA) are measures of pressure and/or volume that estimate the amount of fluid in the entire cardiovascular system at a specific point in time. They are intended to assess volume status and are most useful as “alarm” signals indicating severe hypo- or hypervolemia. Static markers are subject to numerous confounding factors, which limit their precision in the clinical setting. Even measuring baseline CO as a one-time static marker possesses only moderate predictive value to estimate volume status because the cardiac function curve characteristics differ among patients as well as within patients due to continuously changing pathophysiologic conditions. Moreover, any single value does not discriminate where a patient is located on the Frank-Starling curve; on the ascending limb or near the plateau of the curve. Although static markers often help assess a patient's hemodynamic status they do not predict if the patient will be fluid responsive or if vasopressors and/or positive inotropes should be initiated, limiting their value to tailor optimal treatment strategies in the individual patient (45).

Dynamic markers try to determine a patient's location along the curve by inducing a short-term change in preload (e.g., administration of a fluid bolus) and measuring the magnitude of response to the change in preload in real time. Therefore, dynamic markers assess at least two points on the curve to estimate a patient's fluid responsiveness. The magnitude of response is usually measured as a change in CO, SV, or their surrogates such as pulse pressure or other clinical parameters. Dynamic markers do not assess volume status, yet assess fluid responsiveness in order to decrease the incidence of fluid overload, as well as the risk of recurring hypotension.

In general, whether a parameter is considered static or dynamic depends if the parameter is measured at a single static point in time, or if an effect is measured in real time in response to a change in preload (e.g., fluid bolus). This means that most parameters, including clinical and point of care ultrasound (POCUS) can be considered both static and dynamic, depending whether a measurement is taken at a fixed time point or over a period of time in response to a preload challenge. For example, HR in isolation is a static marker, while a change in HR following a fluid bolus is a dynamic marker; the caudal vena cava (CVC) diameter in isolation is a static marker, while the CVC collapsibility index (CVC_CI_), because of heart-lung interactions, is a dynamic marker (see below). Although many parameters can be assessed dynamically in response to a change in preload, many perform poorly because they are influenced by factors independent of preload. These markers are therefore more commonly employed as indicators of circulatory shock or used as static “alarm” markers (e.g., HR, urine output, CVP, etc.; see below). Moreover, many of these typical static markers are not sufficiently sensitive, or their measurement is not sufficiently precise to allow detection of small changes in preload, limiting their use as dynamic markers (26). In contrast, the ideal dynamic marker should be one that truly reflects fluid responsiveness, responding consistently and predictably to an induced change in preload for a specific volume status and not influenced by factors other than preload. To be considered a dynamic marker the parameter assessed should be sensitive and specific for fluid responsiveness, and the parameter should be measured with enough precision to allow for the accurate detection of small changes.

As mentioned, dynamic markers of fluid responsiveness are required to assess the capacity to improve tissue perfusion following an induced change in preload. Given fluid responsiveness aims to precisely titrate fluids, it is more commonly utilized after the initial resuscitation (fluid loading) phase of fluid therapy is complete. However, depending on the level of invasiveness and skill required to assess the dynamic parameter, the applications will vary. For example, the authors commonly assess parameters such as the left atrial to aortic (LA:Ao) ratio and caudal vena cava diameter (CVCd) to assess volume status and the CVC_CI_ to assess fluid responsiveness in the resuscitation phase of fluid therapy.

The preferred techniques used to assess fluid responsiveness may also differ depending on the clinical setting. Some techniques are less precise, yet less invasive, more rapid, and more easily applicable in a less controlled environment, making them perfectly suited for the triage or ER setting (Figure 3). More accurate techniques often require invasive lines, expensive equipment, and patient cooperation, and are typically reserved for a more controlled environment, such as an ICU setting. An overview of the different techniques is presented in the Eisenhower matrix in Figure 3. The following paragraphs will consider several parameters and their value as a static or a dynamic marker.

### Traditional Static Markers

As mentioned above, many parameters can be measured in a static or dynamic fashion. However, the following section discusses clinical exam findings, MAP, shock index (SI), CVP, and lactate as primarily static markers in the clinical setting as evidence suggests they are not as accurate as some markers when assessed dynamically in response to a preload challenge.

#### Clinical Exam Findings

The purpose of the clinical examination in the patient with circulatory failure is to identify patients that are most likely to benefit from IV fluid therapy. The initial assessment of volume status begins with interpretation of clinical signs and symptoms including HR, pulse quality, mental status, jugular vein distention, urine production, skin turgor, temperature, capillary refill time (CRT), and the presence of peripheral edema (17, 25, 39). Although clinical examination findings have been studied and are integral to the assessment of perfusion status and the identification of shock in veterinary patients, studies assessing the accuracy of clinical parameters to specifically estimate volume status and/or volume responsiveness in dogs and cats are very limited (49–51). Research in anesthetized dogs suggests these parameters are not accurate at differentiating fluid responders from non-responders (16–19). A recent study in spontaneously breathing dogs with compromised hemodynamics or tissue hypoperfusion found CRT, presence of pale mucous membranes, HR, weak pulses and serum lactate concentrations all failed to differentiate fluid responders and non-responders (25). Studies in cats evaluating hemodynamic parameters to predict volume status and fluid response are lacking. Similar to canine studies, most human studies demonstrate clinical examination findings, particularly in isolation, fail to discriminate between hypovolemic and normovolemic individuals (36). However, there is some evidence in human ICU patients that using a combination of two or more physiological variables increases the sensitivity and specificity of a diagnosis of impending cardiovascular collapse (52). These examination methods should therefore be used together to identify patients suspected to be in shock and potentially trigger fluid administration (53). In addition, larger prospective clinical studies should be undertaken in veterinary medicine before recommendations regarding the accuracy of traditional static markers for the assessment of volume status and fluid responsiveness can be made in dogs and cats.

During circulatory dysfunction, the compensatory neurohumoral response redistributes blood flow to vital organs by reducing blood flow to non-vital structures such as the skin and mucous membranes. Subjective assessment of the temperature of the extremities (54–56), central to peripheral temperature difference (57), and CRT have been validated and shown to be relatively reproducible in human ICU patients to assess volume status (58). Unfortunately, clinical parameters such as CRT are operator dependent being affected by different duration of pressure, and both ambient and skin temperature (59). As mentioned above, a single study in spontaneously breathing dogs with hemodynamic compromise demonstrated CRT and the presence of pale mucous membranes could not differentiate fluid responders and non-responders (25). Therefore, although these parameters remain integral to the assessment of ECC patients to assess perfusion, they have poor sensitivity and specificity at predicting volume status (36). A change in these clinical parameters in isolation from other parameters should be interpreted cautiously as they may not accurately reflect the volume status of the patient.

Another marker that may be reflective of hypoperfusion is urinary output (36). Hypersthenuric oliguria suggests a hypovolemic state with poor renal perfusion. Oliguria is non-specific and may exist with any form of dehydration regardless of intravascular volume (36). Urine output is influenced by neurohormonal compensatory mechanisms that may preserve or even increase renal blood flow in the face of decreased blood volume and administration of IV fluids could alter renal perfusion by increasing venous congestion (60). Furthermore, there is mounting evidence that the presence of profound intrarenal microcirculatory abnormalities that are not related to global hypoperfusion in resuscitated human patients contribute to acute kidney injury associated with septic shock and major surgery (60). Assessing urine output as a dynamic marker of fluid responsiveness is hampered by the time required for the kidneys to respond to changes in vascular volume, as well as the fact that urine output is influenced by factors other than hemodynamic status. Accordingly, fluid administration does not imply restoration of normal diuresis will be achieved (61), and chasing an increase in urine output with fluid boluses is considered a dangerous strategy by many authors as it can lead to fluid overload.

A specific HR that can be used to guide fluid resuscitation has not been well-studied in either the human or veterinary profession. Tachycardia is an important early sign of shock and is often considered a static marker indicative of hypovolemia (36). Although a dynamic decrease in HR following a fluid bolus suggests some degree of pre-existing hypovolemia, research in dogs suggests the ability of HR to predict fluid responsiveness is variable. A study in anesthetized dogs challenged with 10 ml/kg of synthetic colloid found the percentage decrease in HR was greater in fluid responders than non-responders (17). This is similar to a study in dogs undergoing abdominal surgery that showed a significant decrease in HR following a 10 ml/kg bolus of isotonic crystalloid over 10 min was only detected in fluid responders (18). However, a prospective study in 25 hospitalized dogs found HR decreased significantly in both fluid responders and non-responders following a 4 ml/kg mini isotonic crystalloid bolus, suggesting HR could not differentiate between the two groups (16). Several other studies in spontaneously breathing and anesthetized dogs also found changes in HR failed to differentiate fluid responders and non-responders (15, 19, 28, 32). These findings are similar to human ICU studies that also demonstrate the ability of a change in HR to predict fluid responsiveness is quite variable (62, 63). Unfortunately, assessment of HR as a dynamic marker of fluid responsiveness is limited by its poor specificity, being influenced by pain, anxiety, fever, anemia, and inflammation (36). Furthermore, mechanical ventilation and anesthesia are known to impede neural and humoral control of HR and the administration of positive or negative chronotropic medications further complicates interpretation of a change in HR for the prediction of fluid responsiveness (36). Although changes in HR alone should not be used to predict fluid responsiveness, in conjunction with other parameters, a decrease in HR following fluid administration suggests a positive response to fluid administration (36, 59). In other words a change in HR following a fluid bolus *a posteriori* suggests a positive response, but does not predict the effect of a subsequent fluid bolus.

#### Mean Arterial Pressure

Ensuring MAP remains above a target value is an established goal in maintaining adequate perfusion of vital organs, although it has not been studied extensively as a predictor of fluid responsiveness, particularly in veterinary medicine. Fluid loading is indicated if tachycardia and hypotension are attributed to fluid depletion (36). As an indicator of severity of hypovolemia both hypotension and tachycardia show good predictive value in human patients with hemorrhagic shock (64, 65), however, these are neither sensitive nor specific (36). Changes in MAP can be applied dynamically to assess fluid responsiveness, and an increase in MAP following a fluid bolus is reflective of an increase in CO and a positive hemodynamic response provided vascular tone is intact. A study in dogs presenting to the emergency room with non-cardiogenic hypotension demonstrated normalization of blood pressure following fluid therapy is associated with a favorable prognosis. However, the study was not designed to determine if blood pressure could predict volume status or response to fluid therapy (32). In contrast, when vascular tone is altered, a failure of MAP to increase following therapy does not imply the absence of a positive response. Inversely, MAP can remain unchanged despite an increase in CO because arterial blood pressure is tightly regulated (66). Most studies in anesthetized and spontaneously breathing dogs demonstrate MAP is unable to discriminate between fluid responders and non-responders even in the face of an increase in CO or SV (16–19, 25). The low predictive value of MAP is likely explained by the influence of underlying conditions on systemic vascular resistance (for instance, vasoplegia in sepsis), and individual variation in normotensive values. Furthermore, hypotension can be associated with non-hypovolemic shock states in which fluid resuscitation is contraindicated (e.g., congestive heart failure). Conversely, in some hypovolemic states compensatory mechanisms that increase vascular resistance may be sufficient to preserve MAP (53, 67). The International Consensus Conference on Hemodynamic Monitoring in 2006 found moderate-to-low evidence to implement target blood pressures in the management of shock in humans in the absence of relevant clinical studies (68).

#### Shock Index

Although HR and MAP individually are considered rather poor indices to guide fluid resuscitation, the identification of tachycardia coupled with hypotension should trigger the clinician to strongly consider fluid resuscitation in the absence of cardiac failure (36, 59). The combination of assessing HR and blood pressure in relation to each other has lead to development of the SI, which is the ratio of HR divided by systolic blood pressure (HR/SBP). The range of SI reported in dogs is quite variable and the precise cut off to identify shock is not well-defined (69–71), however a value of ≥0.9 has been associated with the presence of shock and increased mortality in dogs (69–71). Studies investigating the precision of SI to predict IV volume status and fluid responsiveness in veterinary patients are lacking. In people, the SI demonstrates a linear inverse relationship with CO and SV (72), and correlates with the extent of hypovolemia in severely injured patients, as reflected by higher rates of massive transfusion, morbidity, and mortality (73). Therefore, SI may prove to be a sensitive parameter for detection of hypovolemia and can be considered a trigger for fluid loading, although it must be considered in light of clinical findings as it lacks specificity, being increased in cardiogenic and obstructive shock. Moreover, as cats often present with bradycardia in response to shock, the SI requires investigation before it can be used in this species.

#### Central Venous Pressure and Pulmonary Artery Occlusion Pressure

Central venous pressure (CVP) and pulmonary artery occlusion pressure (PAOP) are still two of the most commonly used hemodynamic monitoring tools in the assessment of volume status in human ICU's (26). Central venous pressure is a measure of pressure in the vena cava or right atrium and reflects RAP. PAOP reflects left atrial pressure and is measured by inserting a balloon-tipped, multi-lumen catheter (e.g., Swan-Ganz catheter) into a central vein, and advancing the catheter through the chambers of the right heart to the level of a branch of the pulmonary artery. When the balloon is inflated, the branch of the pulmonary artery is occluded providing a pressure reading that is considered equivalent to the pressure of the left atrium. However, evidence in humans suggests CVP is a poor surrogate for RAP, cardiac filling pressure or preload changes (45). This is because the ventricular pressure-volume curve is not linear and the CVP is affected by alterations in cardiac, lung, and intrathoracic pressures (45). Both CVP and PAOP are invasive, relatively expensive, technically demanding to place, and have been associated with complications. However, given CVP was found to have clinical significance at very low and high values in human ICU patients, if a central line is placed for other reasons, extreme CVP values or consistent trends over time may still help identify states of severe hypo- and hypervolemia (26, 74). Although extreme values may help answer the clinical decision to start or stop fluid therapy, neither CVP nor PAOP can predict fluid responsiveness in people (26, 75). Similarly, evidence suggests CVP is not a good predictor of fluid responsiveness in dogs and performs poorly when compared to dynamic variables (17, 18). In summary, invasive lines should not be placed for the sole purpose of measuring CVP, but can be used when placed for other reasons. If used, CVP should be reserved for the detection of clinically relevant hypovolemia or hypervolemia, as opposed to determining optimal volume status. Given an increasing CVP during preload challenge in humans may have negative predictive power for fluid responsiveness it should not be used to predict or guide fluid resuscitation but may be used as a safety endpoint (26).

#### Blood Lactate

Blood lactate concentration is a downstream marker of tissue perfusion and a hallmark of shock, which is often used as a trigger to initiate and help guide fluid resuscitation (59, 72). However, lactate is non-specific and hyperlactatemia may be associated with any cause of decreased DO_2_ or shock, regardless of volume status (45, 56). It may also be increased secondary to drug administration, decreased metabolism or elimination, or production by bacteria (76). Moreover, hyperlactatemia secondary to an anaerobic cellular metabolism in hypovolemic patients only occurs when a certain threshold of hypoperfusion has been reached; it is not sensitive at accurately identifying mild hypo- or even severe hypervolemia. Although lactate has been studied in veterinary medicine as a prognostic indicator in many disease states, research regarding the ability of lactate to discriminate between fluid responders and non-responders is lacking; a single study in spontaneously breathing dogs failed to demonstrate a difference in lactate between responders and non-responders (15). There is even less research regarding lactate and volume status in cats; it has been shown that hypotensive cats with normal lactate have a greater chance of survival than hypotensive cats with hyperlactatemia (39), however, lactate has not been studied as a hemodynamic marker of volume status or fluid response in cats. There is also recent evidence in the human literature that titrating fluid boluses based on lactate clearance in patients with septic shock may lead to volume overload with an increased risk of organ dysfunction and death (45). Therefore, similar to HR and MAP, lactate should be considered a good “alarm” signal, indicating a degree of urgency to initiate fluid resuscitation in patients suspected to be hypovolemic. Although it is a good marker of perfusion, in isolation lactate may not accurately reflect volume status or always predict fluid responsiveness.

### Traditional Dynamic Markers

#### Fluid Bolus, Mini Bolus, Heart-Lung Interactions, and Passive Leg Raising

In order to dynamically assess any physiologic response to a preload challenge it is necessary to define and standardize what a preload challenge is. The preload challenge is designed to induce a change in preload that subsequently causes a measurable change in a physiologic parameter with minimal risk of causing fluid overload. Currently, a preload challenge may be defined as a traditional fluid bolus, mini fluid bolus, heart-lung interaction, or PLR. In veterinary medicine, consensus on what constitutes a fluid bolus is lacking but 10–20 ml/kg of isotonic fluid administered over 10–15 min is often used. In contrast a mini fluid bolus is typically 3–5 ml/kg of isotonic fluid administered over 1–5 min (14–16, 18, 20, 23, 24, 32–35, 77).

It is also possible to induce changes in preload without physically administering fluids through the principle of heart-lung interactions (45, 78). This can help predict patients that are fluid responsive, prior to fluid administration, and potentially reduce the amount of fluid given to non-responders. The relationship between intrathoracic pressure (ITP), VR and cardiac function forms the basis for understanding heart-lung interactions (Figures 1B,C) (78). It is based on the principle that changes in ITP during the respiratory cycle can be applied to form dynamic tests of fluid responsiveness. During spontaneous ventilation, ITP (the pressure within the pleural cavity) decreases during inspiration (becomes more negative) and increases during expiration. The inverse occurs with positive pressure ventilation (PPV), ITP increases with inspiration and decreases with expiration (29, 78). In a patient receiving PPV, during inspiration there is an increase in ITP and RAP. As VR relies on the difference between MCFP and RAP, it decreases during the inspiratory phase of PPV (Figure 1C) (29, 78). The decrease in VR during inspiration decreases right ventricular filling which subsequently decreases CO from the right ventricle. In addition, there is a direct effect of PPV on the heart during the inspiratory phase, which causes increased right ventricular afterload (79). The effects of this are seen on the left ventricle during expiration, due to pulmonary transit time. Pulmonary transit time means that the respiratory effects of ITP on the right side of the heart are mirrored by the left side of the heart but during the opposite phase of the respiratory cycle (79). Therefore, a decrease in left ventricular preload and decreased left ventricular output will be noted during expiration (30). By manipulating heart-lung interactions though manipulation of ventilator settings it is possible to increase preload and test fluid responsiveness of mechanically ventilated patients before administering any fluids. Alternatively, if positive pressure variables are held constant, serial changes in heart-lung interactions can be assessed in response to a fluid bolus or PLR maneuvers.

In human medicine, the administration of a “virtual fluid bolus” has been described through the application of PLR (80). PLR shifts the venous blood volume from the legs to the central circulation, acting as a reversible preload challenge. As the actual blood volume is unaltered, if the patient fails to respond appropriately or shows signs of volume overload, the “virtual bolus” can be reversed by simply returning the legs to a lower position. A major advantage of the PLR is the fact that it can be assessed using multiple different physiologic parameters, and therefore performed in both mechanically ventilated and spontaneously breathing patients, patients with cardiac arrhythmias, and those receiving low tidal volume ventilation (26). However, if pulse pressure variation (PPV), systolic pressure variation (SPV), stroke volume variation (SVV) and plethysmographic variability index (PVI), are used to assess the response of a PLR, then mechanical ventilation and general anesthesia will be required in most cases (see below). Although appealing, the PLR maneuver is more challenging in awake spontaneously breathing veterinary patients as a change in patient positioning may elicit an adrenergic/sympathic or “white coat response” in many companion animals. Adrenergic/sympathetic responses have been shown to confound changes induced by PLR maneuvers in people (81). The practicality of a PLR maneuver in veterinary medicine is limited to anesthetized swine, which coupled with the difficulty in being able to precisely and rapidly assess a response to a PLR maneuver, limits its application in the companion animal setting (31, 82).

In addition to standardizing and defining the preload challenge it is necessary to choose a physiologic variable that is easily measured and sufficiently sensitive to accurately detect even small changes in preload in order to minimize the risk of volume overload. In other words, for dynamic assessment of fluid responsiveness to be applicable, the assessed parameter must be sufficiently sensitive and precisely measured.

#### Cardiac Output

The reference standard to assess a patient's fluid responsiveness following a preload challenge is CO, which is an important part of tissue DO_2_, along with the oxygen content of arterial blood. Cardiac output is also used to describe the response to increasing preload with the cardiac function curve (Figure 2A). Traditionally CO can be measured using thermodilution, LiDCo (lithium dilution CO), pulse contour analysis, and transthoracic impedance (22). However, these techniques are rarely used in the clinical setting and often reserved for research purposes as they tend to be invasive and require special equipment or training. Newer non-invasive devices based on electrical velocimetry (EV) methods have been experimentally introduced in dogs undergoing cardiovascular and cardiac surgery, and in client owned dogs with pulmonary hypertension and myxomatous mitral valve disease. They have shown promise in measuring CO, SVV, and fluid responsiveness although further research is required to determine the clinical applications of these methods in veterinary patients (83–86).

#### Pulse Pressure, Systolic Pressure, and Stroke Volume Variation

**Figure 4:**
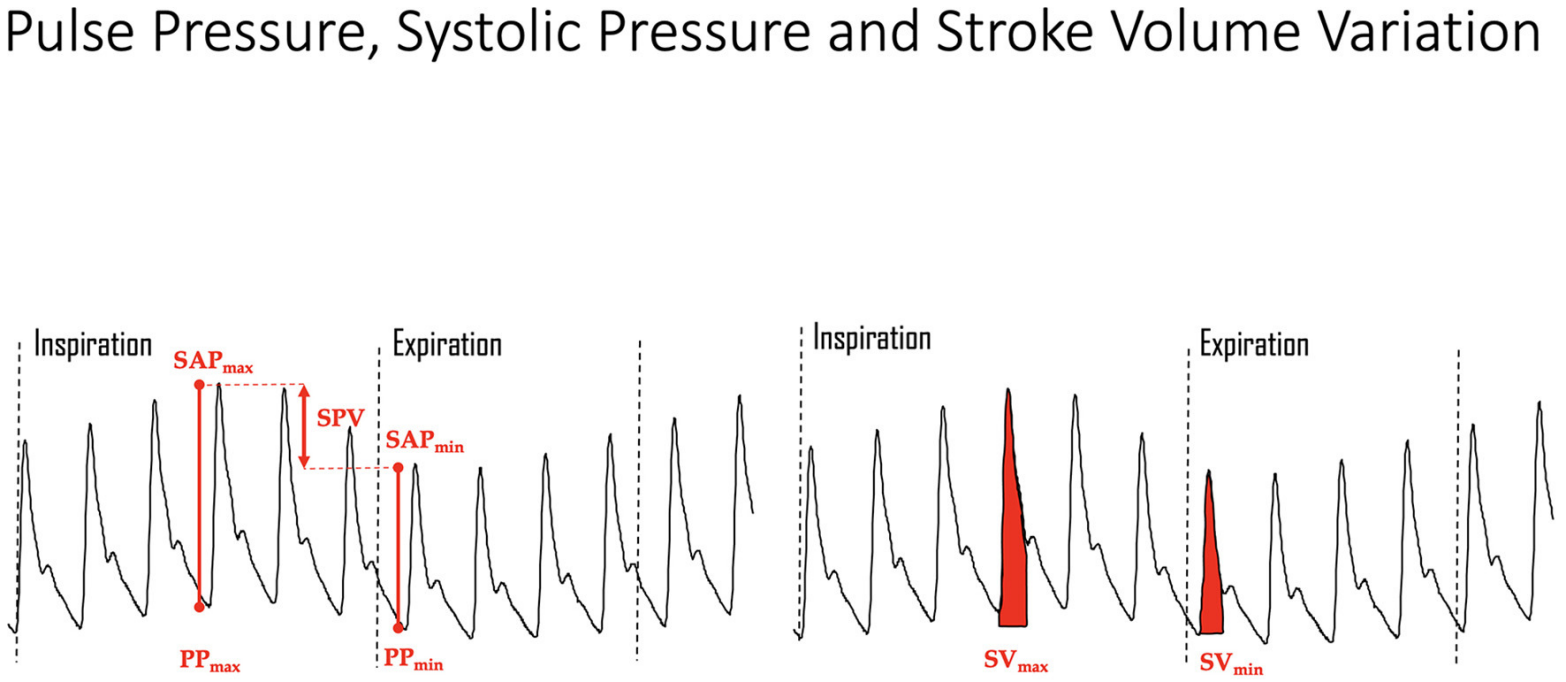
Mechanical ventilation induced variations in the arterial pressure curve showing systolic arterial pressure (SAP) maximum (max) and minimum (min), pulse pressure (PP) maximum and minimum, and stroke volume (SV) maximum and minimum. The greater the degree of hypovolemia, all other variables held constant (e.g., tidal volume, airway pressure, etc.), the greater the change in ventilation induced variations for all parameters. SPV, systolic pressure variation.

Surrogate markers for CO that have been used to dynamically assess fluid responsiveness include SVV, PPV, and SPV (76). These parameters are typically assessed in the anesthetized patient receiving mechanical ventilation and therefore necessitate a highly controlled environment with intensive monitoring. In the mechanically ventilated patient, a controlled change in respiratory pressure induces SVV, PPV and SPV due to the physiology of heart-lung interactions (Figure 4) (37, 76). The magnitude of the change varies with the patient's volume status and fluid responsiveness. A lower position on the Frank-Starling curve will induce larger variations during the respiratory cycle; positive fluid responders will demonstrate higher variations than non-responders (17, 21, 77, 87). In addition to the patient's volume status, the magnitude of change will vary depending on the tidal volume, positive inspiratory pressure, and positive end expiratory pressure chosen to induce a change in preload. Although many ventilator settings that influence the observed response can be controlled, others such as spontaneous respiratory effort, altered chest wall compliance, cardiac disorders (e.g., arrhythmias), right heart failure, and altered intra-abdominal pressures, are more difficult to control. Studies assessing dynamic markers in mechanically ventilated human and veterinary patients often fail to standardize the ventilator setting used to induce a change in preload or fail to agree on the magnitude of response used to define fluid responsiveness, making direct comparison of studies challenging (16, 17, 25, 35, 77, 78, 81, 87). Despite the lack of standardization, several studies have demonstrated the ability of dynamic variables to differentiate fluid responders from non-responders in anesthetized mechanically ventilated dogs. Regarding SVV, a study in healthy dogs that received fluid loading via continuous rate infusions of isotonic crystalloid at 90 ml/kg/h and synthetic colloid at 30 ml/kg/h found SVV of ≥11% to be the optimal threshold to predict fluid responsiveness (sensitivity 100%; specificity 100%) (88). This same study found the optimal threshold for PPV to predict fluid responsiveness was 7% (sensitivity 100%; specificity 100%). A study in client owned dogs undergoing abdominal surgical procedures demonstrated PPV could detect occult hypovolemia and predict cardiovascular response to a fluid bolus of 10 ml/kg isotonic crystalloids over 10 min; a PPV of ≥13% reliably predicted fluid responders in 82.8% of cases (18). Another study in healthy dogs exposed to 6 graded episodes of hemorrhage at 5 ml/kg, followed by 6 graded whole blood transfusions of 5 ml/kg showed moderate accuracy of PPV (sensitivity 71%, specificity 82%) to predict fluid response when a cutoff of ≥16% was used (21). Interestingly the SVV did not change in that study. A study using 10 ml/kg of synthetic colloid administered over 13 min demonstrated PPV is able to predict fluid responsiveness in healthy dogs when cut off values of 11% is used (sensitivity 79%, specificity 80%) (17). A study in client owned dogs undergoing orthopedic or oncologic procedures found PPV could predict fluid response following a 5 ml/kg isotonic crystalloid bolus administered over 15 min when the cut off of ≥15.6% was used (sensitivity 88%, specificity 100%) (19). A study in client owned dogs receiving 15 ml/kg of isotonic crystalloids over 15 min found PPV with a cut off of 15% was ideal to distinguish fluid responders and non-responders (sensitivity 50%, specificity 96%) (24). A 1 ml/kg isotonic crystalloid bolus administered to healthy dogs demonstrated SPV to be reliable predictor of fluid response when a cutoff of >6.7% was used to identify responders (sensitivity, 77.8%, specificity, 93.3%) (23). A study on healthy client owned dogs found the optimal threshold for SPV to detect fluid responders was 4.5% when a 3 ml/kg mini fluid bolus was administered over 1 min (sensitivity of 90%, specificity of 87%) (35). However, results of this study are difficult to compare with others as the reference standard used to define fluid response was a 10% decrease in HR and/or increase in MAP in response to the mini fluid challenge, which as discussed, are not reliable indicators of fluid responsiveness. All other studies used echographic derived calculations of CO and/or SV with increases of >10–15% as the reference standard to define a fluid response, except for three studies that used transpulmonary thermal dilution CO (21, 22, 88). Overall, these studies support the use of dynamic variables to predict fluid response in dogs, however, cut off variables differed significantly between studies, which is likely explained by the lack of standardization regarding the volume, type and rate of the fluid administered as well as ventilator settings used. If one combines the results from veterinary studies, fluid-responsiveness can generally be defined as a variation of ≥10–15% in the dynamic parameter measured. Moreover, the greater the variation, provided other variables remain constant, the more likely the patient will benefit from additional fluid boluses (see the gray-zone approach). Research in cats regarding the ability of SVV, PPV, SPV to detect fluid responders and non-responders is lacking.

#### Plethysmographic Variability Index

Plethysmographic variability index (PVI) is an automatic measure of the dynamic change in perfusion index (PI), as determined by a pulse oximeter, occurring during a complete respiratory cycle. PI demonstrates the pulse strength at the sensor site and represents a relative value of tissue perfusion and blood flow at the site of measurement, which can change based on the patient's clinical status. The pulse oximeter derived pulsatile signal is indexed against the non-pulsatile infrared signal and expressed as a percentage reflecting the amplitude of the pulse oximeter waveform. More precisely, the measurement of PVI involves mathematical calculations using PI which are automatically performed by the internal software installed in the pulse oximeter. The PVI is calculated as [(PImax – PImin)/PImax] × 100 (37) (see Table 1). It is considered a non-invasive alternative to PPV for assessment of fluid responsiveness, with higher variability in the plethsmographic waveform being associated with preload dependence and fluid responders (14, 18, 21, 87, 96). A study investigating PVI in dogs undergoing abdominal surgery demonstrated a positive correlation between PPV and PVI, although a threshold for PVI to identify fluid responders was not established (18). Studies in healthy dogs subject to hemorrhage and transfusion of removed blood, as well as synthetic colloids demonstrated PVI had moderate to good ability to predict a positive fluid response with cut off values of 9.3–12% (sensitivity 78–86%, specificity 70–72%) (21, 22). Because PVI is dependent on pulse oximetry readings, it is subject to the same limitations as pulse oximetry; skin pigmentation, ambient light, motion artifact, vascular tone and peripheral perfusion are confounding factors that may prevent accurate values from being obtained (82, 87, 97). Although it does not require an invasive line, given the incidence of confounding factors likely to be encountered in hypovolemic patients it remains to be determined whether PVI is clinically applicable in veterinary patients with circulatory shock. PVI is also dependent on heart lung interactions and therefore subject to the same limitations as PPV, SVI, and SVV. There are also species differences that can affect pulse oximetry readings, which may subsequently influence PVI calculations in veterinary patients if human monitors are used (97).

**Table 1:** Formula and reported threshold values to discern fluid responders from non-responders.

**Parameter**	**Abbreviation**	**Formula**	**Human threshold**	**Canine threshold**
Systolic pressure variation	ΔSP	ΔSP = SP_max_ – SP_min_	dDown > 5 mmHg (89)	dUp > 4 mmHg (35)
Pulse pressure variation	PPV	$\frac{\left(\text{P}{\text{P}}_{\text{max}}-\text{P}{\text{P}}_{\text{min}}\right)\times 100}{\left(\text{P}{\text{P}}_{\text{max}}+\text{P}{\text{P}}_{\text{min}}\right)/2}$	13.7% (90) 6.5–17% (91)	7 (71)−9.3% (17) 13% (18)−15.6% (92)
Stroke volume variation	SVV	$\frac{\left(\text{S}{\text{V}}_{\text{max}}-\text{S}{\text{V}}_{\text{min}}\right)\times 100}{\left(\text{S}{\text{V}}_{\text{max}}+\text{S}{\text{V}}_{\text{min}}\right)/2}$	10–22% (19)	11% (22)
Plethysmographic variability index	PVi	$\frac{\left(\text{P}{\text{i}}_{\text{max}}-\text{P}{\text{i}}_{\text{min}}\right)\times 100}{\text{P}{\text{i}}_{\text{max}}}$	14% (93) 9.5–19.0% (94)	11% (17)−13% (18)
Caudal vena cava collapsibility index	CVC ci	$\frac{\text{CV}{\text{C}}_{\text{max}}-\text{CV}{\text{C}}_{\text{min}}}{\text{CV}{\text{C}}_{\text{max}}}$	50% (95)^*^	30% (21)^**^

*dDown, decrease in systolic pressure; dUp, increase in systolic pressure; max, maximal value obtained during a respiratory cycle; min, minimal value obtained during a respiratory cycle*.

* *Values higher than 50% are strongly associated with hypovolemia rather than indicative of fluid responsiveness*.

** *Mean value in healthy normovolemic dogs*.

### Point of Care Ultrasound

Similar to several parameters already discussed, assessment of point of care ultrasound (POCUS) parameters can be assessed in a static fashion to estimate volume status or applied dynamically to assess fluid responsiveness. POCUS for volume status and fluid responsiveness includes both cardiac and vascular components.

#### Cardiac POCUS

**Figure 5:**
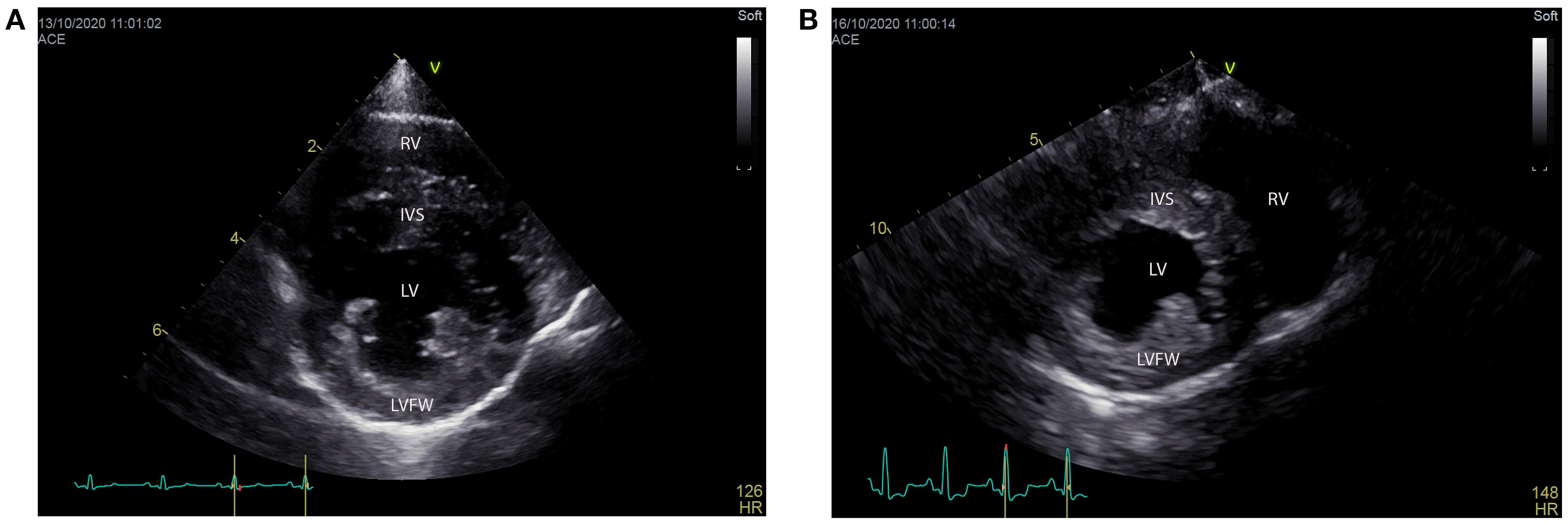
**(A,B)** Right parasternal short axis views from a normal (left) and hypovolemic (right) dog. Note how the left ventricular lumen (LV) appears smaller than the lumen of the normovolemic dog and both the intraventricular septum (IVS) and left ventricular free wall (LVFW) appear thicker. RV, right ventricle.

Cardiac POCUS, also referred to as abbreviated echocardiography, is commonplace in many human ICU settings, predominantly because of its rapid, non-invasive, real time ability to assess patient volume status (98). Clinical research demonstrates cardiac POCUS applied to human patients with indeterminate volume status results in a change in therapeutic management and improved medical decision making (99). Estimation of volume status predominantly focuses on the subjective assessment of cardiac contractility, ventricular lumen size, ventricular wall thickness, and atrial lumen size, all of which require minimal training to learn (98). Similar to humans, dogs and cats with clinical signs of hypovolemia may develop smaller left ventricular and left atrial lumen sizes, and thicker left ventricular walls, referred to as pseudohypertrophy (100–102) (Figures 5A,B). Studies in cats, dogs and people suggest these changes are proportional to the severity of hypovolemia and reversible following successful volume replacement (98–103). In human patients with normal clinical parameters, cardiac POCUS may detect smaller left ventricular cavity and atrial size, indicating volume status is suboptimal (48, 52). Preliminary research in dogs suggests cardiac POCUS may have a higher sensitivity for detection of changes in volume status when compared to clinical parameters (100), although the number of canine studies was small and further research is recommended to corroborate these findings. In an experimental study in cats, volume depletion to the level of a 7–10% body weight reduction resulted in the cardiac POCUS findings of decreased ventricular and atrial lumen size, while left atrial and ventricular lumen size increased following volume administration (101).

**Figure 6:**
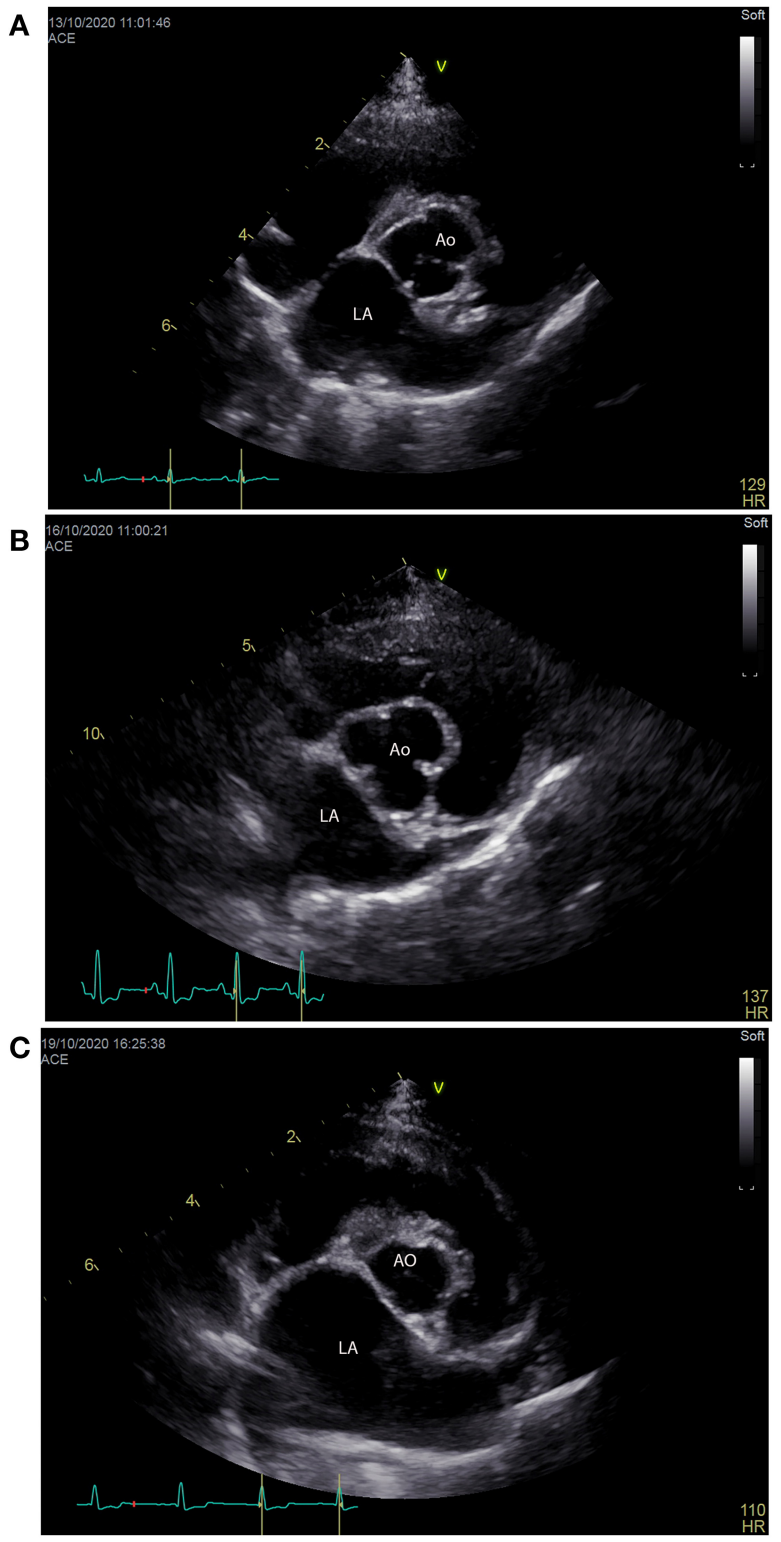
**(A–C)** Right parasternal short axis views showing the left atrial (LA) size compared to the aorta (Ao) size from 3 different dogs that were normovolemic **(A)**, hypovolemic **(B)** and hypervolemic **(C)**. Note the size (width) of the left atrium is only slightly larger than the aorta in a normovolemic patient **(A)**, equal to slightly smaller than the aorta in a hypovolemic patient **(B)** and significantly larger than the left atrium in a patient that is hypervolemic **(C)**.

Left atrial size is probably the easiest clinical cardiac POCUS parameter to assess for volume status estimation in dogs and cats (Figures 6A–C). It is non-invasive, inexpensive, and repeatable, requiring minimal training (104). Either short or long axis right parasternal views can be used to assess left atrial size. In both dogs and cats the normal LA:Ao ratio varies from 1 to 1.5 when measured via the right parasternal short axis window (105, 106). Alternatively, subjective estimation of the number of times the (area of the) aorta can be fit within the left atrial lumen has also been evaluated in cats. In a healthy cat, when looking at the short axis view of the left atrium, if the area of the left atrium is more than 2.5 times the area of the aorta, the left atrium should be considered enlarged (107). The upper reference range for the left atrial width in the long axis view of cats is 16 mm (or up to 18.5 mm in large breed cats such as Norwegian Forest cats or Maine Coons) (108). Although a decrease in left atrial lumen size in a patient with clinical signs of hypovolemia is strongly suggestive of a hypovolemic state, obstructive disease cannot be ruled out. For example, studies in healthy dogs with experimentally induced pericardial effusion developed echographic signs of pseudohypertrophy, including a small left atrium (109). Conversely, an increased left atrial lumen size may occur secondary to hypervolemia, congestive heart disease, or dilated cardiomyopathy.

In addition to changes in lumen size, the ventricular wall thickness should be considered relative to the dimensions of the left ventricular lumen in dogs and cats (100, 101). Although not specific, both thin or “stretched” ventricular walls and thick ventricular walls in diastole can be indicative of hypervolemia and hypovolemia, respectively. However, given thin walls may also be a consequence of dilated cardiomyopathy or regional lesions (e.g., myocardial infarction) and myocarditis, and ventricular hypertrophy can lead to thick ventricular walls, interpretation of findings in light of the entire clinical picture is imperative when assessing volume status.

Subjective and objective assessment of contractility of the left ventricle can also be assessed in relation to volume status, with increased contractility being seen in response to compensatory shock states and as an adaptive response to ventricular filling (110, 111). However, confounding factors such as hypertrophic cardiomyopathy, or even stress or pain via their effect on sympathetic tone can result in increased contractility decreasing the specificity of cardiac POCUS contractility assessment to detect hypovolemic states (110, 111). Although cardiac POCUS appears promising in veterinary medicine for assessment of volume status and can act as a trigger to start or discontinue fluid resuscitation, clinical studies validating its use and accuracy are lacking.

Measurement of left ventricular end-diastolic area (LVEDA) has been used for assessment of volume status in the human critical care setting due to its simplicity of measurement (112). It is measured at the level of the papillary muscles in the parasternal short axis window at end-diastole by tracing the endocardial border (113). A small LVEDA (<10 cm^2^ in humans) suggests significant hypovolemia, while an enlarged LVEDA (>20 cm^2^ in humans) suggests volume overload (114). Obliteration of the left ventricular cavity would be seen in severe hypovolemia (115). It should be kept in mind that LVEDA is considered a static marker and prone to the same limitation as other static markers of volume status. Concentric left ventricular hypertrophy and constrictive pericarditis may also cause a small LVEDA independent of volume status (112, 116). Furthermore, specific cut offs for LVEDA have not been established (112) and a recent meta-analysis demonstrated failure of the LVEDA to predict volume responsiveness in mechanically ventilated human patients (117).

At a more advanced level CO can be directly calculated based on the cross-sectional area of the descending aorta and the left ventricular outflow tract volume time integral (LVOT VTI), which has been used to predict fluid responsiveness in both humans and dogs (15, 116). The product of these factors is equal to the column of blood that is ejected from the heart during each contraction. Similar to previously discussed parameters, heart-lung interactions will induce a change in LVOT VTI. Alternatively, changes in LVOT VTI can be measured dynamically before and after a mini fluid bolus or PLR in humans, similar to other traditionally measured dynamic markers. A recent study demonstrated the accurate assessment of fluid responsiveness in conscious dogs using the LVOT VTI (15). Unfortunately, sonographic assessment of LVOT VTI requires a much higher skill level compared to other POCUS measurements. A small error in the calculation of the aortic surface, as well as a slightly altered angle when assessing the LVOT VTI by Doppler ultrasound can induce changes in measurements that are greater than the expected change induced by a mini-bolus or heart-lung interactions.

#### Caudal Vena Cava Diameter and Collapsibility Index

Inferior vena cava diameter (IVC_d_) has become well-established in many human ECC settings as a means of assessing volume status (118). In companion animals, caudal vena cava (CVC) assessment has been described at the suprailiac (kidney), the right intercostal (transhepatic) and the subxiphoid (diaphragmatic) level (119). Research suggests all three sites can easily be assessed in dogs, although inter-rater variability at the subxiphoid view is higher than the suprailiac or right intercostal approaches (119). In contrast to human medicine where the IVC is commonly used in the ECC setting (98), recent surveys demonstrate many veterinarians lack confidence and require further training to feel comfortable evaluating the caudal vena cava (CVC) (120, 121). Assessing the CVC may also be challenging in patients with abdominal discomfort.

**Figure 7:**
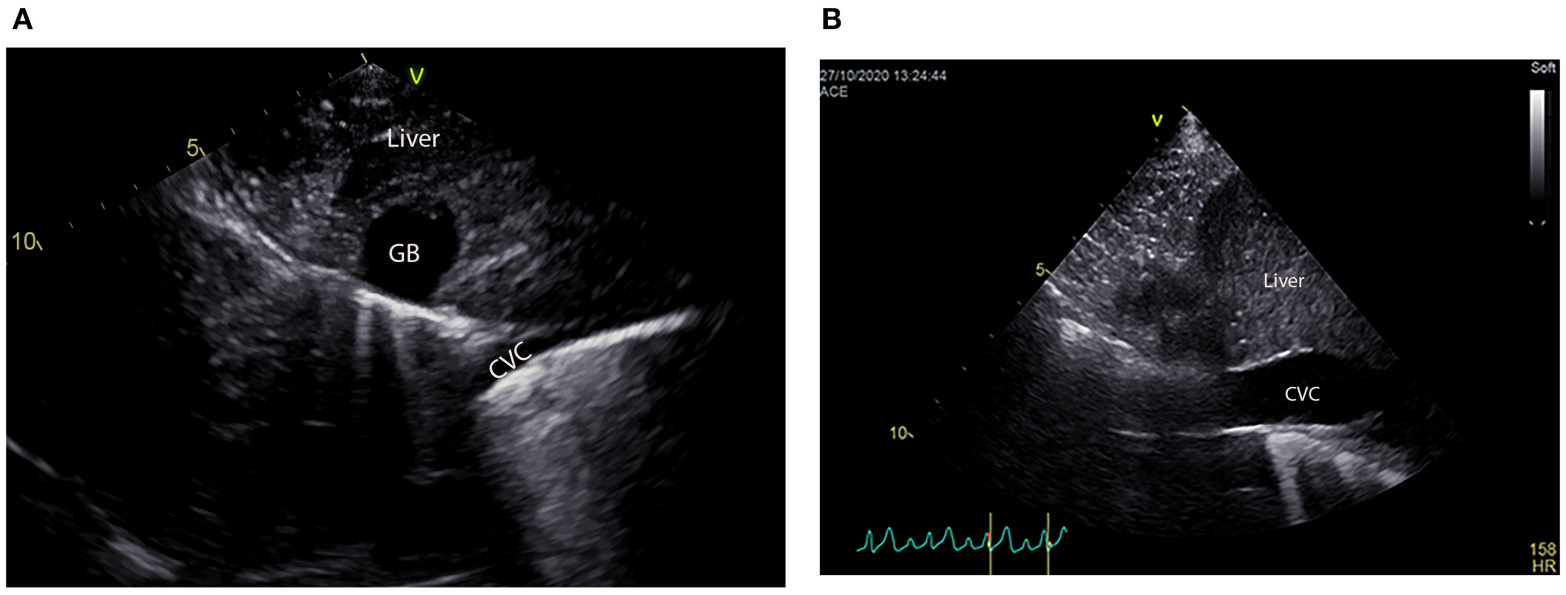
**(A, B)** Long axis views of the caudal vena cava (CVC) as it crosses the diaphragm assessed at the subxiphoid site of 2 dogs that were hypovolemic **(A)** and hypervolemic **(B)**. Note the CVC is very narrow or “flat” in the hypovolemic dog and quite wide or “fat” in the hypervolemic dog. There is also a suspicion of free abdominal fluid between the liver lobes in the hypervolemic dog, although free fluid cannot be differentiated from artifact in this single still image. Free abdominal fluid would not be unexpected in dogs with hypervolemia and a more thorough point of care ultrasound evaluation would be recommended to further assess this finding.

Studies in human medicine demonstrate that a decreased, or small/flat, IVC diameter (IVC_d_) correlates with hypovolemia and poor IVC dilation following fluid resuscitation suggests inadequate intravascular volume, independent of arterial blood pressure. Similar to humans, CVC diameter (CVC_d_) has been shown to correlate with volume status and CVP in dogs (122). An experimental model of clinically undetectable hypovolemia in dogs created through blood donation (roughly 8% blood volume) demonstrated mixed results ranging from no significant change to a mild yet significant decrease in the CVC_d_ (123–125). Inversely, in dogs with chronic degenerative mitral valve disease (DMVD), increasing American College of Veterinary Internal Medicine (ACVIM) disease stage is associated with higher CVC_d_ secondary to fluid retention and hypervolemia (126) (Figures 7A,B).

In addition to the correlation of the CVC_d_ with blood volume, the thin elastic nature of the CVC also makes it a responsive and dynamic vessel. The size and geometry fluctuate in response to relative and absolute intravascular volume changes depending on the cardiac and respiratory cycle (see Supplementary Videos 1–3). Breathing changes intrapleural pressures, generating heart-lung interactions, thereby influencing the intravascular volume within the thorax and abdomen. This change in size of the CVC between inspiration and expiration, referred to as CVC collapsibility, is calculated as a collapsibility index (CVC_CI_). The CVC_CI_ expresses the percentage change in the diameter of the CVC during the respiratory cycle based on the following formula: CVC_CI_ = (CVC_d_ max – CVC_d_ min)/CVC_d_ max (see Table 1).

The IVC_CI_ has shown promise in human medicine to predict fluid responsiveness (127). A “fat” IVC, subjectively defined as wide IVC with an IVC_CI_ < 50% is associated with a high CVP secondary to hypervolemia, congestive heart disease or cardiac tamponade. Inversely, a flat IVC, defined as a narrow IVC with an IVC_CI_ > 50% is correlated with a low CVP and is indicative of hypovolemia. Mean IVC_CI_ values reported in healthy adult humans are 47.3 ± 8.9% (128). An advantage of the IVC_CI_ in humans is the fact it can be assessed in patients undergoing positive pressure ventilation, and in critically ill spontaneously breathing patients (129, 130). Although the threshold to distinguish fluid responders from non-responders in spontaneously breathing patients varies between studies (see gray-zone approach), an IVC_CI_ ≥ 48%, predicts fluid responsiveness with a sensitivity of 84% and a specificity of 90% (129). Despite these promising findings, IVC assessment is influenced by factors such as cardiac function, respiratory effort, intra-abdominal pressure, and pressure artifact (131).

There are limited veterinary studies examining the use of CVC_CI_ in dogs or cats to assess fluid responsiveness. Blood donation in dogs appears to be associated with an increased CVC_CI_ (124). More advanced ACVIM stage of DMVD in dogs also is associated with a decreased CVC_CI_ (126). A canine study indicated CVC_CI_ can predict fluid responsiveness under controlled circumstances in anesthetized and mechanically ventilated dogs (23). Although these findings suggest the CVC_CI_ is a promising marker of fluid responsiveness in companion animals, further research is required before guidelines on its application can be recommended. A very small clinical study (*n* = 27) in spontaneously breathing dogs with compromised hemodynamics or tissue hypoperfusion suggested that CVC_CI_ can accurately predict fluid responsiveness, although the authors also concluded research is necessary to extrapolate their results to larger populations of hospitalized dogs (25).

#### Lung Ultrasound and Other POCUS Findings Suggestive of Volume Overload

Despite significant advances in the understanding of the physiology behind fluid therapy, hemodynamically unstable human and veterinary ECC patients often receive IV fluids without defining clear end points of fluid resuscitation or start points for fluid removal (132). Failure to identify end points of fluid resuscitation may lead to fluid overload and increased EVLW (45, 132–134). Evidence suggests that increased EVLW can be detected with lung ultrasound (LUS) through the identification of increased B lines: comparison of a reference standard for detection of EVLW and LUS for detection of increased B lines shows there is a direct correlation between the two (135–137), and evidence in humans shows the sensitivity and specificity of LUS to identify pulmonary edema (defined as increased B lines) is as high as 97 and 95%, respectively.

**Figure 8:**
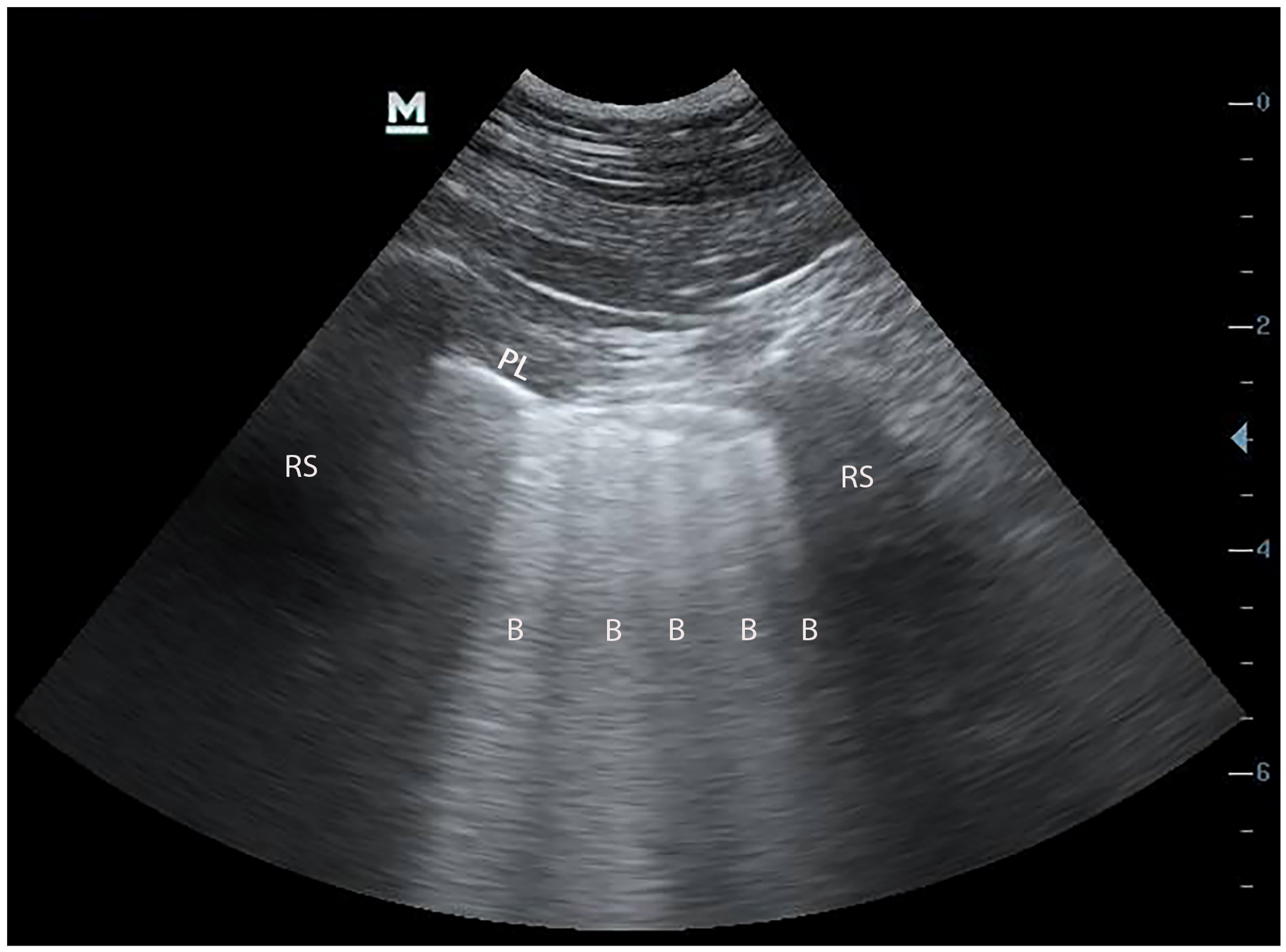
Still ultrasound image obtained during pleura and lung ultrasound (PLUS) in a dog with “wet lungs.” The presence of >3 B lines is considered “wet lung” and indicates increased extravascular lung water. Although there are multiple causes of “wet lung,” fluid overload should be suspected in a patient that has received fluid therapy fluid, particularly if the patient progresses from dry to wet lung on serial PLUS evaluation.

B lines are lung surface artifacts that result from a proportional increase of fluid in the interstitium and/or alveoli, which creates a high fluid-air impedance gradient between the fluid and air within the lung (138). These B lines appear sonographically as hyperechoic (white) vertical narrow (individual) to broad (coalescing) bands that extend vertically from the lung surface and move synchronously with lung sliding (Figure 8) (139, 140). In healthy veterinary patients it is possible to detect B lines in up to 31% of patients, however it is more common to find only a single isolated B line at 1 location on either hemithorax, or two B lines at a single location when scanning the lung surfaces of dogs and cats (141–144). Although 3 B lines at a single location has been described in healthy cats and dogs, this number of B lines in a single sonographic window is rare and should not occur at multiple lung locations over the same hemithorax (142, 143). The appearance of >3 B lines in a single lung ultrasound window, or a serially increasing number of B lines relative to base line is suggestive of “wet lungs” (142, 143, 145). However, as increased B lines are not specific and may result from numerous alveolar interstitial pathologies (e.g., fluid overload, left sided congestive heart failure, pulmonary contusions, aspiration pneumonia, acute respiratory distress syndrome, fibrosis, neoplasia, etc.) it is important to identify the underlying cause to direct appropriate diagnostics and therapy (146–150).

The correlation between increased B lines and EVLW has led to the development of the fluid administration limited by lung sonography (FALLS) protocol in human ICU settings to help physicians detect a safety threshold for fluid resuscitation in patients presenting in shock (151). The FALLS protocol is based on the principle that LUS can accurately detect increased B lines (“wet lung”) in patients with increased EVLW, which can be used to help guide management of patients presenting with unexplained circulatory shock. Based on the FALLS protocol patients with uncomplicated hypovolemic shock that have not yet received fluid boluses will have an absence to very few B lines, or “dry lungs,” throughout all sonographic lung fields. Tailored fluid therapy in these patients should correct hypovolemia without leading to an increase in B line numbers or “wet lungs.” The FALLS protocol will also detect most patients that present with cardiogenic shock and left-sided congestive heart failure as these patients typically are in a state of volume overload at presentation and will likely have “wet lungs” on initial LUS evaluation. Successful therapy of left sided congestive heart failure should result in a serial decrease in the number of B lines detected on LUS (145, 152). Finally, patients with sepsis and distributive shock are more likely to develop “wet lungs” during fluid loading, despite continuing signs of circulatory failure. If a patient progresses from “dry” to “wet lungs” at any point in time further patient evaluation and re-assessment of the fluid therapy plan is indicated.

**Figure 9:**
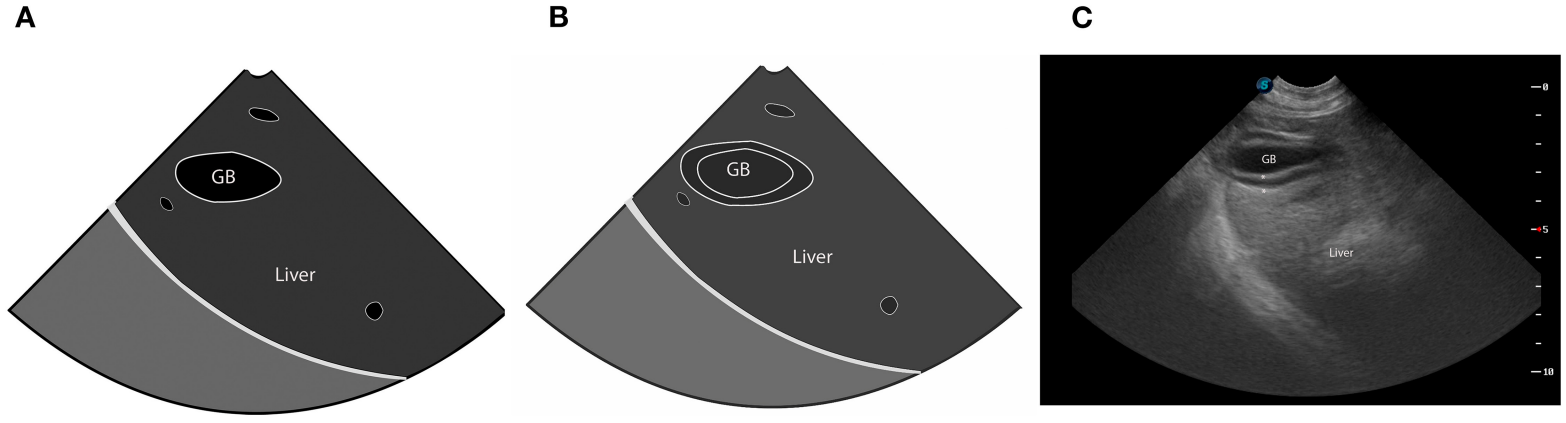
**(A–C)** Schematic images of a normal (left) gall bladder, gall bladder wall edema also referred to the halo sign (middle), and still ultrasound image of the halo sign (right). With gall bladder wall edema there is a thickening of the gall bladder wall but also a “striated” appearance that arises when anechoic fluid (black) separates the mucosal and serosal surfaces of the gall bladder wall (white). The serosal and mucosal surfaces of the gall bladder wall are indicated by an asterix in the still image. GB, gall bladder.

Additional POCUS findings that may be identified in patients developing volume overload secondary to excessive fluid administration include fluid accumulation in body cavities and development of gall bladder wall edema, often referred to as a gall bladder “halo sign” (Figure 9) (122). Although the development of a gall bladder halo sign is non-specific and commonly associated with anaphylaxis (153), the appearance of a halo sign during fluid loading can be suggestive of hypervolemia. If the halo sign occurs secondary to hypervolemia with increased right atrial pressure, it is often associated with other sonographic findings suggestive of volume overload, such as an enlarged CVC (see above) and/or the appearance of pleural effusion and/or free abdominal fluid (122).

In summary, POCUS findings suggestive of volume overload include enlarged left atrial and ventricular lumen size, thinning of the ventricular walls, a distended CVC with a decreased CVC_CI_, increased number of B lines, gall bladder wall edema and body cavity fluid accumulation. As these findings are not specific, it is important to interpret POCUS findings in light of the entire clinical picture.

## Conclusions

Optimizing individual fluid therapy is complex with very little veterinary evidence available to guide clinical decision-making. However, a greater knowledge and understanding of the differences between volume status and fluid responsiveness, and the factors influencing both allow the clinician to interpret clinical findings with greater confidence. The decisions regarding estimation of fluid volume and fluid responsiveness vary depending on the clinical scenario and how well the patient's environment can be controlled. POCUS provides rapid real time patient assessment that can guide volume status and fluid responsiveness regardless of the setting. The clinician should however consider a gray-zone approach in answering the question of volume status and fluid responsiveness to minimize clinically relevant hypo- or hypervolemic states and ensure optimal fluid management in critical care patients. Within the gray-zone approach more precise titration of fluids becomes paramount and fluid responsiveness should be assessed through techniques that minimize the risk of volume overload; small IV bolus administration, and/or assessment of parameters that respond to heart-lung interactions in the absence of administering fluids.

## Author Contributions

Both authors listed have made a substantial, direct and intellectual contribution to the work, and approved it for publication.

## Conflict of Interest

The authors declare that the research was conducted in the absence of any commercial or financial relationships that could be construed as a potential conflict of interest.

## Supplementary Material

Supplementary Video 1 Caudal vena cava (CVC) changes in a dog with hypovolemia. Note the change in diameter of the CVC between inspiration and expiration (the CVC collapsibility index) is easily visible and subjectively changes by about 90%. Smaller pulsatile fluctuations in the CVC as a results of the cardiac cycle are also visible. The degree of change in the CVC diameter visible during the cardiac and respiratory cycles is consistent with hypovolemia and a patient that is likely fluid responsive.

Supplementary Video 2 Caudal vena cava (CVC) changes in a dog with increased right atrial pressures. Note there is minimal change in diameter of the CVC between inspiration and expiration (the CVC collapsibility index), and the smaller pulsatile cardiac fluctuations in the CVC are absent. The lack of visible change in the CVC diameter during the cardiac and respiratory cycles in this dog is consistent with increased right atrial pressures (e.g., iatrogenic fluid overload, pericardial effusion, right sided heart failure etc.). Further work up is recommended to determine the cause.

Supplementary Video 3 Caudal vena cava (CVC) changes in a cat that accidently received a blood transfusion over 10 min. Note there is minimal change in diameter of the CVC between inspiration and expiration (the CVC collapsibility index), and the smaller pulsatile fluctuations in the CVC as a results of the cardiac cycle are absent. The lack of visible change in the CVC diameter during the cardiac and respiratory cycles is consistent with increased right atrial pressures, and in this case secondary to volume overload from rapid blood transfusion therapy. Pleural effusion is also visible which was not present when the cat presented, which further supports volume overload. The cat was successfully treated for iatrogenic volume overload with furosemide.
